# Dynamic Nonlinear Behavior of Ionic Liquid-Based Reservoir Computing Devices

**DOI:** 10.1021/acsami.2c04167

**Published:** 2022-07-26

**Authors:** Takuma Matsuo, Dan Sato, Sang-Gyu Koh, Hisashi Shima, Yasuhisa Naitoh, Hiroyuki Akinaga, Toshiyuki Itoh, Toshiki Nokami, Masakazu Kobayashi, Kentaro Kinoshita

**Affiliations:** † Department of Applied Physics, Graduate School of Science, Tokyo University of Science, Katsushika, Tokyo 125-8585, Japan; ‡ Device Technology Research Institute, National Institute of Advanced Industrial Science and Technology, Tsukuba, Ibaraki 305-8565, Japan; § Toyota Physical and Chemical Research Institute, Nagakute, Aichi 480-1192, Japan; ∥ Center for Research on Green Sustainable Chemistry, Faculty of Engineering, Tottori University, Koyama, Tottori 680-8552, Japan; ⊥ New Value Creation Office, NAGASE & CO., LTD., Nihonbashi, Tokyo 103-8355, Japan

**Keywords:** ionic liquid, faradaic current, reservoir computing, liquid/solid interface, electrochemical reaction

## Abstract

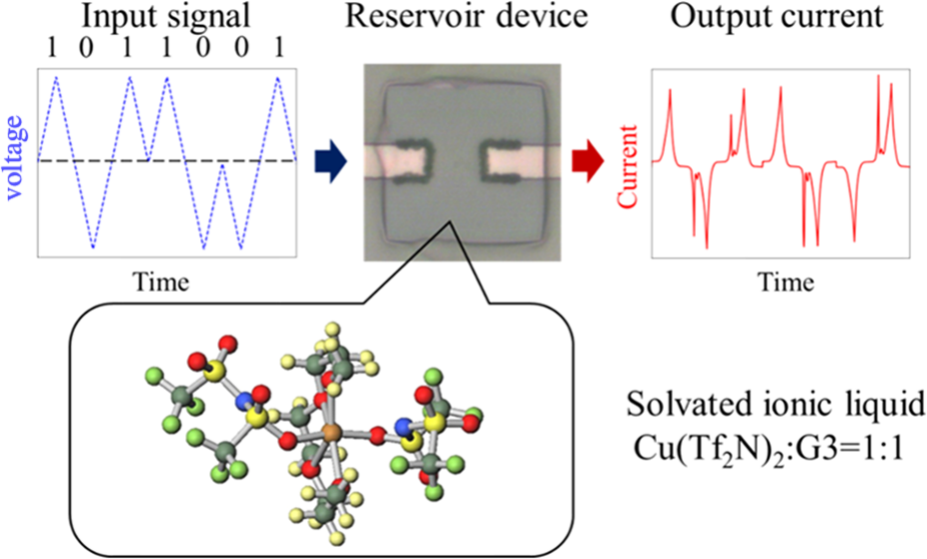

Herein, a physical reservoir device that uses faradaic currents generated by redox reactions of metal ions in ionic liquids was developed. Synthetic time-series data consisting of randomly arranged binary number sequences (“1” and “0”) were applied as isosceles-triangular voltage pulses with positive and negative voltage heights, respectively, and the effects of the faradaic current on short-term memory and parity-check task accuracies were verified. The current signal for the first half of the triangular voltage-pulse period, which contained a much higher faradaic current component compared to that of the second half of the triangular voltage-pulse period, enabled higher short-term memory task accuracy. Furthermore, when parity-check tasks were performed using a faradaic current generated by asymmetric triangular voltage-pulse levels of 1 and 0, the parity-check task accuracy was approximately eight times higher than that of the symmetric triangular voltage pulse in terms of the correlation coefficient between the output signal and target data. These results demonstrate the advantage of the faradaic current on both the short-term memory characteristics and nonlinear conversion capabilities and are expected to provide guidance for designing and controlling various physical reservoir devices that utilize electrochemical reactions.

## 1 Introduction

Owing to the accelerating development of the Internet of Things (IoT) technology, neural networks have been increasingly used for information processing in many applications. However, the deep neural network (DNN) learning process is costly in terms of both time and computational resources, particularly when DNNs are combined with edge devices such as sensors.

**Figure 1:**
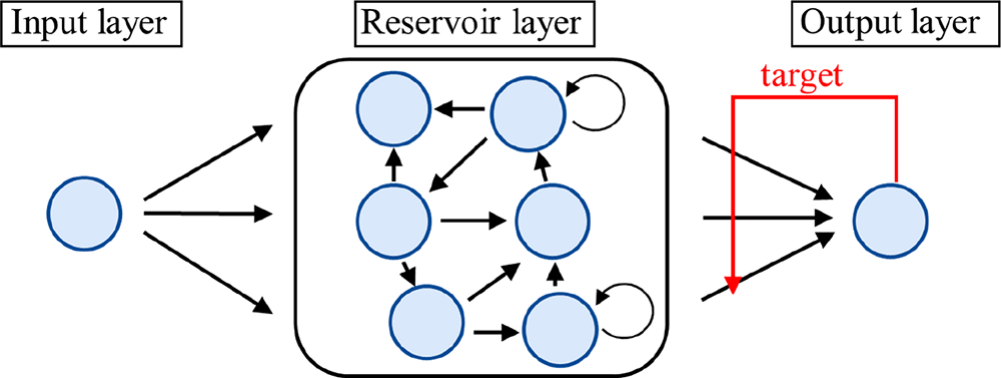
Schematic of reservoir computing. Circles depict the nodes in each layer. Black arrows depict the weights between the nodes, while closed arrows represent time correlation in each node. The red arrow from the output node represents the update of the weight values between the reservoir and output layers.

Recently, physical reservoir computing (PRC), which implements the computational model shown in Figure 1 in a physical device, has attracted considerable attention.^1,2^ PRC uses the dynamics of a physical device as the reservoir layer (i.e., reservoir device (RD)). In this model, only the weights between the reservoir and output layer are updated during the learning process. The reduction in the numbers of rewriting and storage of weights leads to more energy-efficient information processing compared with conventional DNNs. Feature extraction abilities of RD are related to how the input signal is transformed into a nonlinear output signal, as well as the short-term memory for the input history.^3^ Various RDs have been proposed based on physical phenomena such as dielectric relaxation in ferroelectrics, spin relaxation, and consistency in lasers.^4−8^ Furthermore, metal redox reactions in solid materials, including WO_*x*_, Ag-doped SiO_2_, Ag_2_S, poly(vinylpyrrolidone)-coated Ag nanowires, and liquid solutions, are also applicable in RDs.^9−14^ The dependence of information-processing performance on the shape of the output current curve has been investigated in detail.^4,13^ In particular, complex nonlinear current responses to voltage inputs have been demonstrated to enhance the information-processing abilities of ferroelectric field-effect transistors (FeFETs).^4^ Moreover, complex nonlinear currents produced by redox reactions in liquid solutions have proven to be advantageous for PRC.^13^ However, the impact of nonlinear current waveforms on PRC performance has not been fully elucidated. Cyclic voltammetry (CV) is an electrochemical measurement method that can produce complicated current curves depending on the sweep voltage. For example, the voltage level and sweep rate can modify the current waveform by changing the extent and velocity of electrochemical reactions. To reliably perform CV measurements under varied voltage-sweep conditions, the materials used for RD devices must be electrically tolerant not to be decomposed by voltage application.^15^ Therefore, the redox reactions that occur at the metal electrode/ionic liquid (IL) interface were used to investigate the systematic connection between the operating characteristics of the RD and PRC information-processing performance. ILs have a relatively large potential window and can be used as reliable reaction fields during electrical measurements.^16^ Furthermore, the material properties of ILs can be systematically controlled via the selection and combination of anions and cations that comprise IL as well as by dissolving various metal salts in the IL as many metal ions can exist stably in an IL. Previously, we successfully used metal redox reactions to control the data volatility of conducting-bridge memory-based memristors^17^ using the copper ion valence in ILs.^18^ However, the IL cations that form the inner Helmholtz layer on the anode surface prevented Cu ions from approaching the anode. The solvated IL, a composite of Cu(Tf_2_N)_2_ and 2,5,8,11-tetraoxadodecane (G3) [Cu(Tf_2_N)_2_/G3 = 1:1], allowed easy access of Cu^2+^ to the anode, as the ions were coordinated by electrically neutral G3 molecules.^19^ In addition, the coordinated structure allowed for high Cu concentrations in the form of the Cu(Tf_2_N)_2_-G3 composite (Cu-G3) compared to Cu(Tf_2_N)_2_-doped [bmim][Tf_2_N], where the saturated concentration of Cu(Tf_2_N)_2_ was ∼0.4 mol/L.

Herein, we developed RDs with tunable properties that exploit the advantages of Cu-G3. High-speed CV measurements were performed using a microfabricated device with Cu-G3 as a reaction field. The current response arising from the redox reactions of metals was evaluated by applying a voltage pulse. The pulse width was set to several hundred milliseconds, which is similar to the timescale of biological electroencephalographic reactions.^20^ Furthermore, the relationship between the metal redox reactions and the PRC information-processing abilities was investigated by changing the output current dataset used for the PRC learning process as well as the voltage level of the RD input signal. The faradaic current significantly improved the accuracy of PRC performance, confirming the influence of redox reactions.

## 2 Experimental Section

### 2.1 Characterization of the IL

The moisture content in Cu-G3 was quantitatively measured by Karl Fischer titration (KFT) (CA-200 titrator and VA-230 vaporizer, Nittoseiko Co., Ltd.). Cu-G3 was characterized by Raman spectroscopy (NRS-5500 Raman spectrometer, JASCO Corporation) and X-ray photoelectron spectroscopy (XPS) (ULVAC-PHI, Quantera II).

The vaporization coulometric Karl Fischer method was selected for KFT because the chemical reaction between the Cu ions in Cu-G3 and iodine ions in the Karl Fischer reagent was thought to negatively influence the titration results. Approximately 50 μL of Cu-G3 was sealed in a vial and annealed at 200 °C for 3 min to vaporize the remaining water. The vaporized water was transferred to the titrator using a nitrogen carrier gas, and the water content was evaluated.

Raman spectroscopy measurements were conducted at 23 °C in air. The Cu-G3 droplet on the Pt (100 nm)/Ta (1 nm)/SiO_2_/Si substrate was measured at an excitation wavelength of 532 nm.

XPS measurements were performed using an Al Kα monochromatic source with a photon energy of 1486.6 eV. The Cu-G3 droplet on the SiO_2_/Si substrate was used for XPS measurements. The detection angle was 45°, corresponding to a detection depth of approximately 4–5 nm. The detection area had a diameter of 100 μm. The Cu 2p_3/2_, N 1s, and O 1s XPS profiles were analyzed in detail.

### 2.2 Device Fabrication

**Figure 2:**
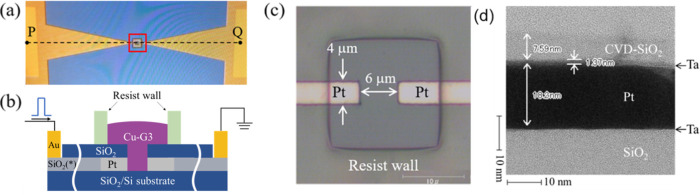
(a) Top-view photograph of the reservoir device and (b) schematic cross section along the black dotted line P–Q in panel (a). SiO_2_ marked by an asterisk in (b) is drawn to explain that the sidewall of the Pt electrode excepting the tip section is covered by the SiO_2_ layer. (c) Optical microscopic image corresponding to the inside of the red frame in panel (a). The electrode width is 4 μm and the distance between the electrodes is 6 μm. (d) Cross-sectional TEM image of the Pt electrode covered by the CVD-SiO_2_ layer.

Figure 2a shows a top view of the fabricated device. The cross section along the dotted line PQ in Figure 2a is schematically depicted in Figure 2b. An enlarged view of the area inside the red frame in Figure 2a is presented in Figure 2c. This device is referred to as “IL-reservoir” throughout the manuscript. Figure 2d shows a cross-sectional transmission electron microscopy (TEM) image of the SiO_2_ and metal layers. The thicknesses of Pt, Ta, and SiO_2_ were determined from the TEM images. The total thickness of Pt and Ta was ∼19.5 nm, although the interface between Pt and Ta remained unclear because of the similar atomic numbers of Pt and Ta. The expected locations of the top and bottom Ta layers are indicated by black arrows. The SiO_2_ layer thickness was determined to be ∼7.59 nm. A three-layer Ta (1 nm)/Pt (20 nm)/Ta (1 nm) structure was prepared on a thermally oxidized Si substrate by magnetron sputtering. Subsequently, a SiO_2_ (20 nm) layer was deposited via chemical vapor deposition (CVD) at 350 °C. Ta (1 nm) on Pt acts as an adhesion layer. A SiO_2_ layer was deposited on top of the metal to apply an electric field between the electrodes. Input and output electrodes with a width of 4 μm were patterned using conventional photolithography and dry-etching processes. The gap between the edges of the electrodes was 6 μm. Contact pads consisting of Au (100 nm)/Ti (10 nm) were prepared via electron-beam deposition to improve the electrical contact of the electrode. The resist wall structure around the input and output electrode edges was patterned by photolithography, followed by annealing at 120 °C for 10 min in the air to remove water, diluent solvent, and alkaline-solubilized constituent residues after development and rinsing. The area inside the resist wall structure was 18 × 26 μm^2^, and the height of the resist wall was ∼2 μm. This resist wall confines the IL by suppressing IL migration and assists in determining the IL volume. A microdroplet of Cu-G3 was placed into the resist wall region using a W needle attached to a high-precision positioner and high-magnification optical microscope.

### 2.3 Operando Microscopy for IL-Reservoir Device

For operando (real-time) observation of the IL**-**reservoir using a high-magnification optical microscope, a semiconductor analyzer (Keysight B1500A) was used to measure the direct current *I*–*V* characteristics of the device during operation. Additional details are provided in the Supporting Information.

### 2.4 Time-Series Data Processing

**Figure 3:**
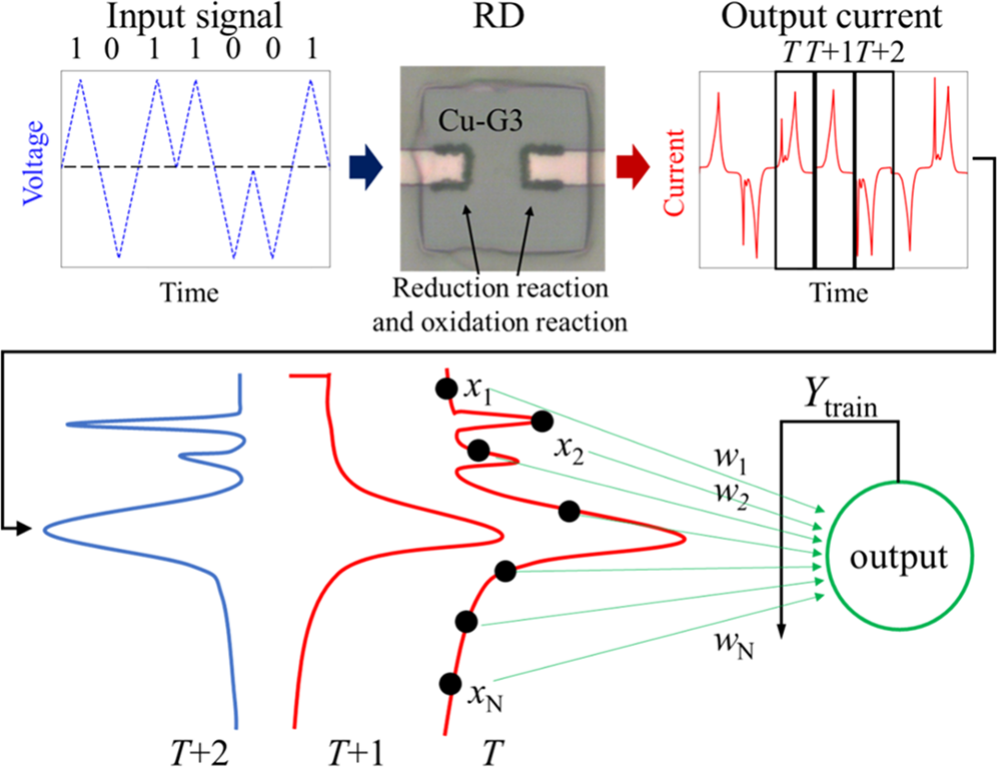
Schematic diagram of the signal processing flow for the physical reservoir calculation. The input signal was a triangular pulse. Output current values *x*_1_, *x*_2_, ..., *x_N_* at each time step (..., *T*, *T* + 1, *T* + 2, ...), which were generated by the redox reactions at the Cu-G3/electrode interface, were obtained to input *N* virtual nodes. Learning to determine values of *w*_1_, *w*_2,_ ..., *w_N_* was conducted by linear regression with *Y*_train_ as the training data.

Figure 3 shows a schematic of the physical RD model using an IL**-**reservoir. A synthetic time-series signal consisting of randomly selected binary data (1 and 0) was input to the device as triangular-shaped voltage pulses (TVPs) using a Keysight B1530A waveform generator/fast measurement unit. The signs of TVP for 1 and 0 were positive and negative, respectively.

Short-term memory (STM) and parity-check (PC) tasks were used to evaluate the PRC performance.^21^

Short-term memory characteristics of the IL**-**reservoir were evaluated based on STM tasks. The training data for the STM tasks are expressed as follows
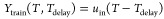
1where *u*_in_ (*T*) is a random input signal (1 or 0) in time step *T*. Namely, the input signal that is *T*_delay_ time step before is used as training data.

The nonlinearity of the IL-reservoir response was evaluated based on PC tasks. The training data for the PC tasks can be expressed as follows
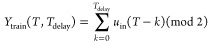
2In addition, for simplicity, the STM and PC tasks for *T*_delay_ = *i* are represented as STM_i and PC_i, respectively, where *i* is an integer. The current value for each time step was acquired as a virtual node, and the number of virtual nodes for each time step was 100.^22,23^ The number of injected voltage pulses, which corresponds to the number of time steps, was also 100. Stochastic gradient descent (SGD) was used to update the weights.^24^ The 100 output data points were divided into 70 and 30 pulses, which were used as training and prediction data, respectively. The current value was normalized using the absolute value of the largest of all output current values. Mini-batch learning was used for the training process, and weight updating was repeated every 10 datasets.

## 3 Results and Discussion

### 3.1 Characterization of Cu-G3

**Figure 4:**
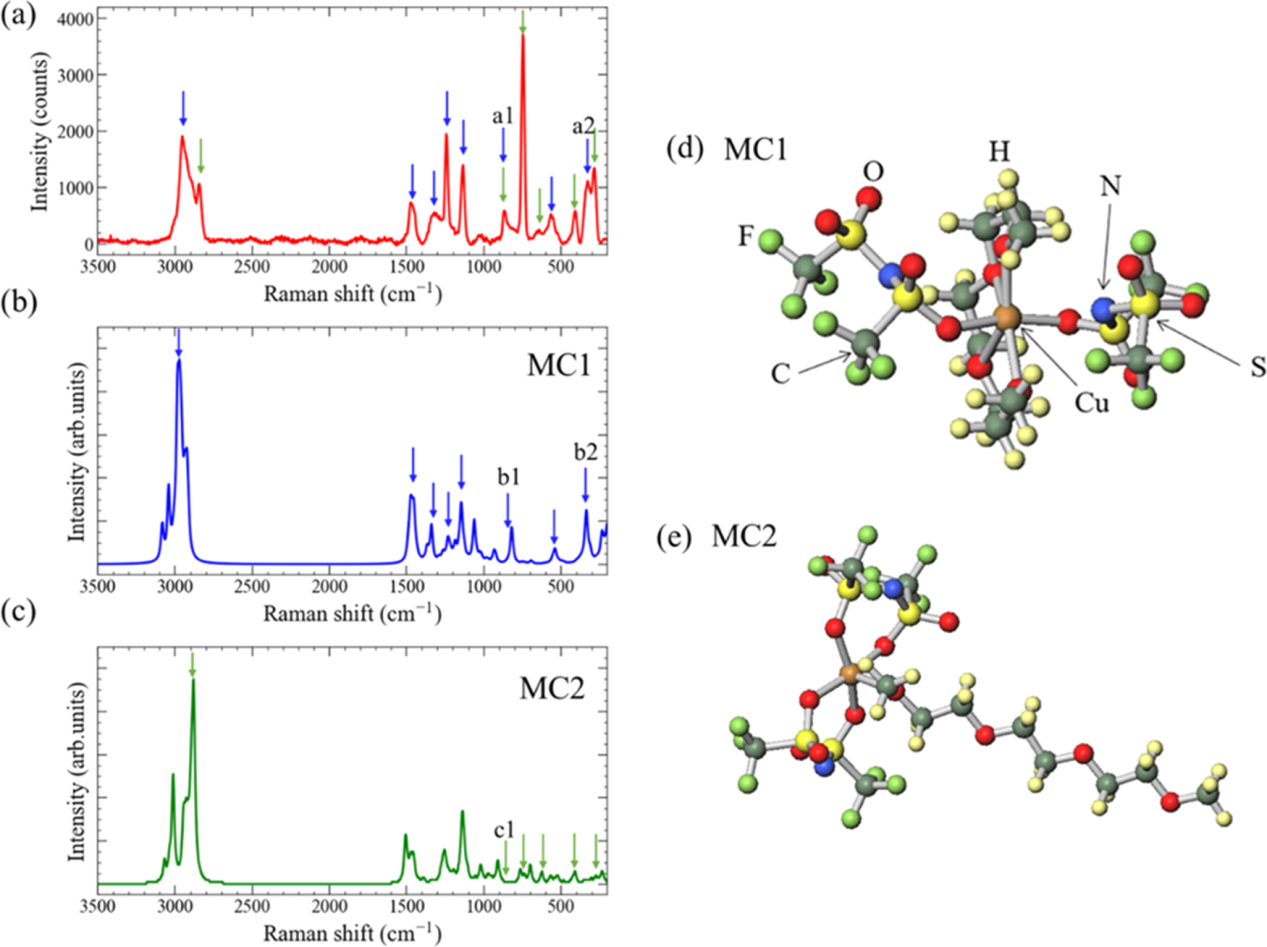
(a) Experimental and (b, c) calculated Raman spectra for Cu-G3; (d, e) optimized molecular structure (MC1 and MC2)^19^ used for the Raman spectral calculations in panels (b) and (c), respectively. Blue and green arrows in the Raman spectra in panels (a)–(c) were assigned to the molecular structure in panels (d) and (e). Light yellow, green, blue, red, light green, yellow, and orange spheres correspond to H, C, N, O, F, S, and Cu. Peaks a1 and a2 in panel (a) correspond to the stretching vibrations of the Cu–O chemical bond between the Cu ions in Cu(Tf_2_N)_2_ and O ions in G3. The experimental peak a1 can be explained by the calculated peaks b1 and c1, while the experimental peak a2 can be explained by the calculated peak b2.

The water content in Cu-G3 was 9.6 wt %, as determined by KFT. Although Cu-G3 underwent a freeze-drying dehydration process immediately after synthesis, moisture absorption from the air likely increased the water content. Furthermore, relatively large amounts of water can be contained in ionic liquids with metal cations.^25^Figure 4 shows the experimental (Figure 4a) and calculated (Figure 4b,c) Raman spectra of Cu-G3. Two molecular configurations are present in Figure 4d,e, represented as MC1 and MC2,^19^ respectively, and were used to calculate the Raman spectra in Figure 4b,c, based on their optimized structures. Most of the peaks in the calculated and experimental Raman spectra corresponded to the two molecular configurations. The experimental Raman peaks indicated by the blue arrows in Figure 4a coincide with the calculated Raman peaks indicated by the blue arrows in Figure 4b. In addition, the Raman peaks indicated by the green arrows in Figure 4a coincide with the calculated Raman peaks indicated by the green arrows in Figure 4c. The peak observed at ∼870 cm^–1^ represented as a1 in Figure 4a originates from two types of Cu–O vibration modes, one in MC1 and the other in MC2 (b1 in Figure 4b and c1 in Figure 4c). The calculated Raman spectra in Figure 4b,c show Raman activity plotted as a function of the Raman shift, where the Raman activity is generally unproportional to the experimental Raman intensity. Therefore, although the peak intensities of the calculated Raman activity of b1 in Figure 4b and c1 in Figure 4c were quite small, the vibration mode corresponding to these activities is likely the origin of the experimental Raman peak a1 in Figure 4a. In contrast, peak a2 originated from the stretching vibration of the Cu–O chemical bond between the Cu ions in Cu(Tf_2_N)_2_ and O ions in G3 in MC1 (b2 in Figure 4b). Subsequently, it was experimentally confirmed that the Cu ions in Cu-G3 chemically interacted with G3, although Cu-G3 contained a relatively large number of water molecules. The details of each Raman peak origin are summarized in the Supporting Information.

**Figure 5:**
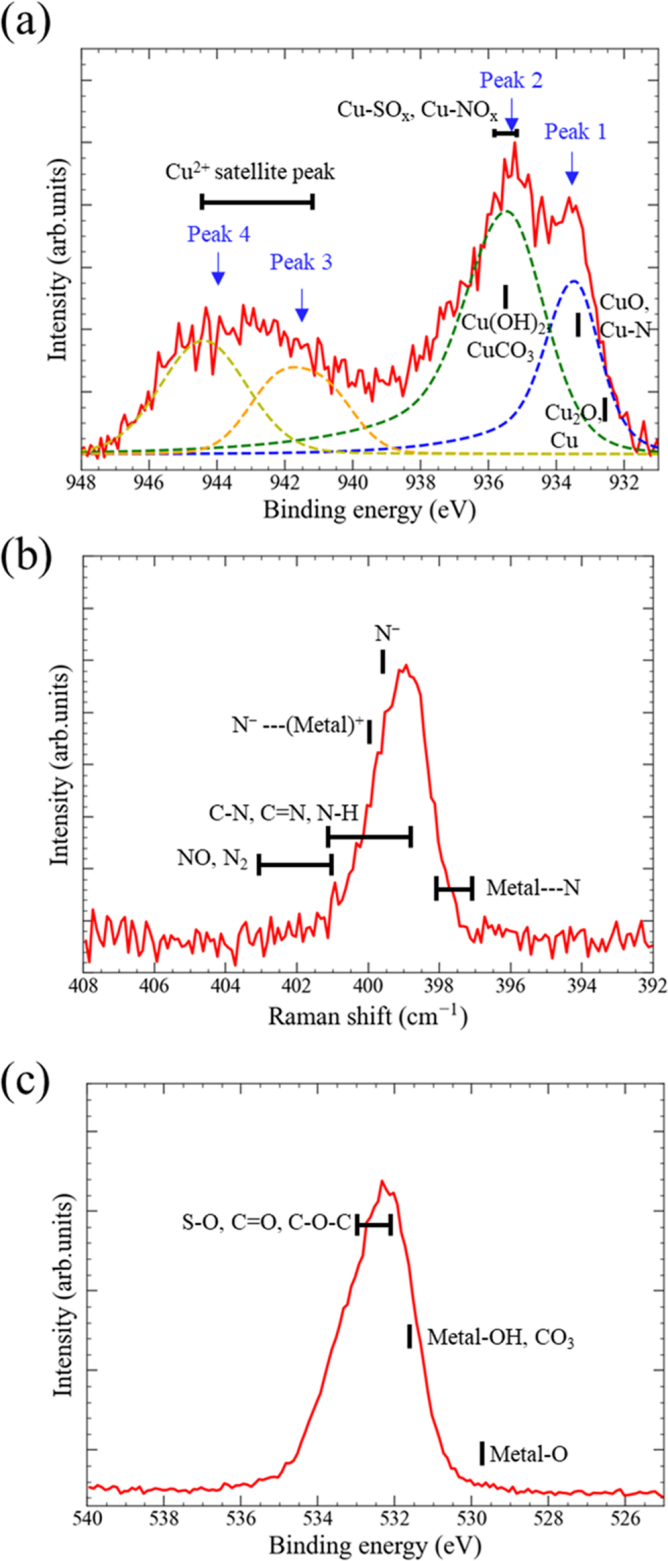
(a) Cu 2p_3/2_, (b) N 1s, and (c) O 1s XPS profiles measured using a Cu-G3 droplet on the SiO_2_/Si substrate. Typical peak positions observed in some Cu-containing compounds and Cu metals for Cu 2p_3/2_, N 1s, and O 1s XPS profiles are also shown.^26,27^ The peak deconvolution results are also plotted with dotted lines.

The signature peaks attributed to the chemical interaction between Cu cations and light elements in G3, including C, O, and N, were observed in the X-ray photoelectron spectroscopy (XPS) profiles (survey spectra for a general overview of elemental species in Cu-G3 are provided in the Supporting Information). Figure 5a shows the Cu 2p_3/2_ XPS profile for Cu-G3 together with the reference peak positions for Cu compounds, including CuNO*_x_*, CuSO*_x_*, and CuCO_3_.^26^ In addition, the peak deconvolution results are plotted in Figure 5a with dotted lines. As shown in Figure 5a, the Cu 2p_3/2_ XPS profile of Cu-G3 exhibited four peaks. Peaks 3 and 4 were observed in the binding energy (BE) range from 940 to 950 eV, corresponding to the shake-up satellite structure, indicating the presence of Cu^2+^ in Cu-G3. Peaks 1 and 2 in the BE range from 930 to 940 eV are the main peaks in the Cu 2p_3/2_ XPS profile. Peak 2 at a BE of ∼935.5 eV is likely derived from Cu^2+^ and correlated with the above satellite structure because the peak position primarily shifted toward higher BEs when the valence of the metal cation increased. The peak 2 position in Cu-G3 was similar to those of CuNO*_x_* and CuSO*_x_*, indicating that the electrons are likely shared by N, S, and O in [Tf_2_N^–^]. These characteristics were also observed in Cu (Tf_2_N)_2_/[bmim][Tf_2_N]. However, peak 1 at a BE of ∼933 eV was associated with a lower Cu valence state than that of Cu^2+^. Because the peak 1 position is clearly at a higher BE compared to that of metallic Cu, this peak was reasonably assigned to Cu^+^. It is well-known that the peak 1 position is nearly identical to that arising from the chemical bonding between Cu^+^ and N^–^.^27^ Therefore, the Cu 2p_3/2_ spectra indicate that the Cu cations in Cu-G3 can exist stably in both the Cu^2+^ and Cu^+^ states, which bind to [Tf_2_N^–^]. Chemical bonding between Cu^+^ and N^–^ is also indicated in the N 1s XPS profile shown in Figure 5b. Chemical bonding between Cu, C, and O is also implied by the O 1s XPS profiles in Figure 5c. The chemical bonds of C–O–C in the BE range from 532 to 533 eV, as well as those of CuCO_3_ at a BE of ∼531.5 eV, were detected in this spectrum. Given the Raman spectra in Figure 4, this CuCO_3_ signal was attributed to the interaction between the O and C ions in G3 and Cu cations. It should be noted that a reversible change in the external appearance of the Cu-G3 droplet on the SiO_2_/Si substrate was observed when the droplet was placed under ultrahigh vacuum for XPS measurement, as shown in the Supporting Information. The external appearance change was attributed to the evaporation of water in Cu-G3. Therefore, the influence of water evaporation on the chemical bonding state in Cu-G3 under vacuum was considered negligible.

### 3.2 IL-Reservoir Mechanism

**Figure 6:**
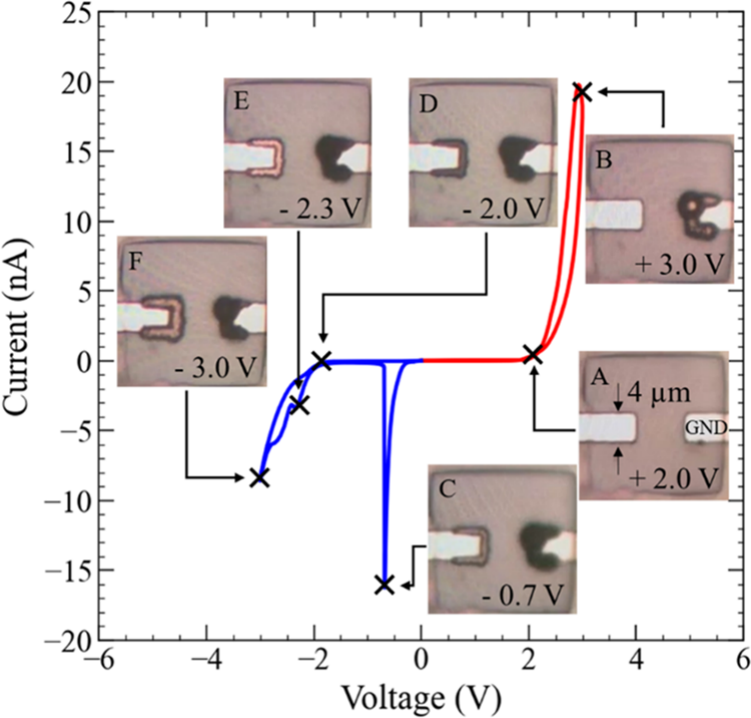
*I*–*V* characteristics of the IL-reservoir prepared using Cu-G3. Insets (A) and (B) are the operando optical microscope images near the input and output Pt electrodes when the voltage is swept in the positive direction. The insets (C–F) are the operando optical microscope images when the voltage is swept in the negative direction. The voltage values in the insets correspond to the cross marks on the *I*–*V* curve. In insets (B–F), Cu deposits on the Pt electrodes can be observed.

Figure 6 shows the CV curves measured using the IL-reservoir in the as-fabricated state. First, the voltage was swept in the positive direction (0 → +3.0 → 0 V) and then in the negative direction (0 → −3.0 → 0 V). Figure 6 insets are real-time optical microscopy images taken concurrently with CV curve measurements. The voltage values corresponding to each optical microscope image (Figure 6A–F) were +2.0, +3.0, −0.7, −2.0, −2.3, and −3.0 V. The voltage-sweep speed was 50 mV/s. As shown in the insets, the Pt-electrode appearance did not change below +2.0 V. Increasing the positive voltage to +3.0 V caused a rapid current increase (evident in the CV curve) and Cu deposition on the Pt electrode on the right, which was grounded during the measurement (Figure 6 inset B). When the CV curve was subsequently measured for a voltage sweep in the negative direction, a sharp current peak appeared at −0.7 V, attributed to redox reactions on both of the Pt electrodes. These reactions included dissolution of Cu that was once deposited during the positive voltage application from the anodic Pt electrode (right) and Cu deposition on the cathodic Pt electrode (left; Figure 6 inset C). From Figure 6 insets C and D, when the negative voltage was increased from −0.7 to −2.0 V, the change was negligible except for a slight darkening of the Cu deposit on the left Pt electrode. When the negative voltage was further increased to −2.3 V, the brightness of the Cu deposit on the left Pt electrode, which is likely to be the metallic luster of copper, notably increased (Figure 6 inset E). Finally, the amount of Cu deposited on the left Pt electrode increased when a negative voltage of −3.0 V was applied (Figure 6 inset F), which occurred simultaneously with the increasing current, as exhibited by the CV curve. It should be noted that the voltage required for Cu deposition on the right Pt electrode in the first voltage sweep (+2.0 V) was approximately three times larger than that required for Cu deposition on the left Pt electrode in the subsequent voltage sweep (−0.7 V). If Cu deposition during the first voltage sweep is caused by identical electrochemical reactions as the subsequent voltage sweep, this result can be attributed to a cathodic reaction (Cu deposition), as described in detail below. In the as-fabricated IL**-**reservoir, no Cu was deposited on either Pt electrode. Therefore, the occurrence of some kind of oxidation reaction on the left Pt electrode is essential for initiating Cu deposition (reduction reaction) on the right Pt electrode. One possible origin of this reaction is water electrolysis contained in the IL and/or an oxidization reaction involving oxygen in air. This is underscored by Cu deposition on the Pt electrode in the first voltage sweep that was not observed when the CV measurement was conducted under vacuum (see the Supporting Information for further details), implying that the atmosphere significantly influences the device operation of the IL**-**reservoir, similar to Li-ion air batteries.^28^ However, upon initiation of the second voltage sweep in the negative direction, Cu was already present on the right Pt electrode. Therefore, it was determined that Cu dissolution occurs as the oxidation reaction on the right Pt electrode instead of water electrolysis, which is necessary to induce Cu deposition on the left Pt electrode. As previously reported, the Cu dissolution reaction occurred at a lower voltage compared to that of water electrolysis.^29^ Accordingly, Cu deposition on the left Pt electrode occurred at a relatively low voltage in the second sweep. In Li-G3, increased water content reportedly causes ligand exchange from G3 to H_2_O, resulting in a relative increase in the Li^+^ diffusion constant compared to that before H_2_O addition.^30^ In other words, the Li^+^ diffusion in Li-G3 was enhanced in the presence of water. This enhancement of metal-ion diffusion by water is analogous to the enhanced Cu ion migration mediated by water in memristive devices based on metal–insulator–metal structures.^31^ For Cu-G3, the Cu cation diffusion enhancement would be reasonably expected, although the Cu cations in Cu-G3 interacted with G3, as indicated by the Raman spectra.

The complex brightness changes in the Cu deposit on the left Pt electrode observed under the negative voltage sweep in Figure 6 are as follows: initial darkening (C → D), brightening (D → E), and second darkening (E → F) of the Cu-containing deposit. These changes are attributed to the formation and breakdown of the highly resistive passive state of Cu, along with a subsequent increase in the Cu surface roughness owing to Cu deposit thickening. In addition, since the Cu^+^ and Cu^2+^ states coexist in Cu-G3 (as determined by the XPS measurements), electron transfer between these two states may also contribute to current flow (Figure 6).^32^ The retention characteristics of the Cu deposits on the Pt electrode formed under different voltage conditions are shown in the Supporting Information.

### 3.3 Pulse-Height Dependency

**Figure 7:**
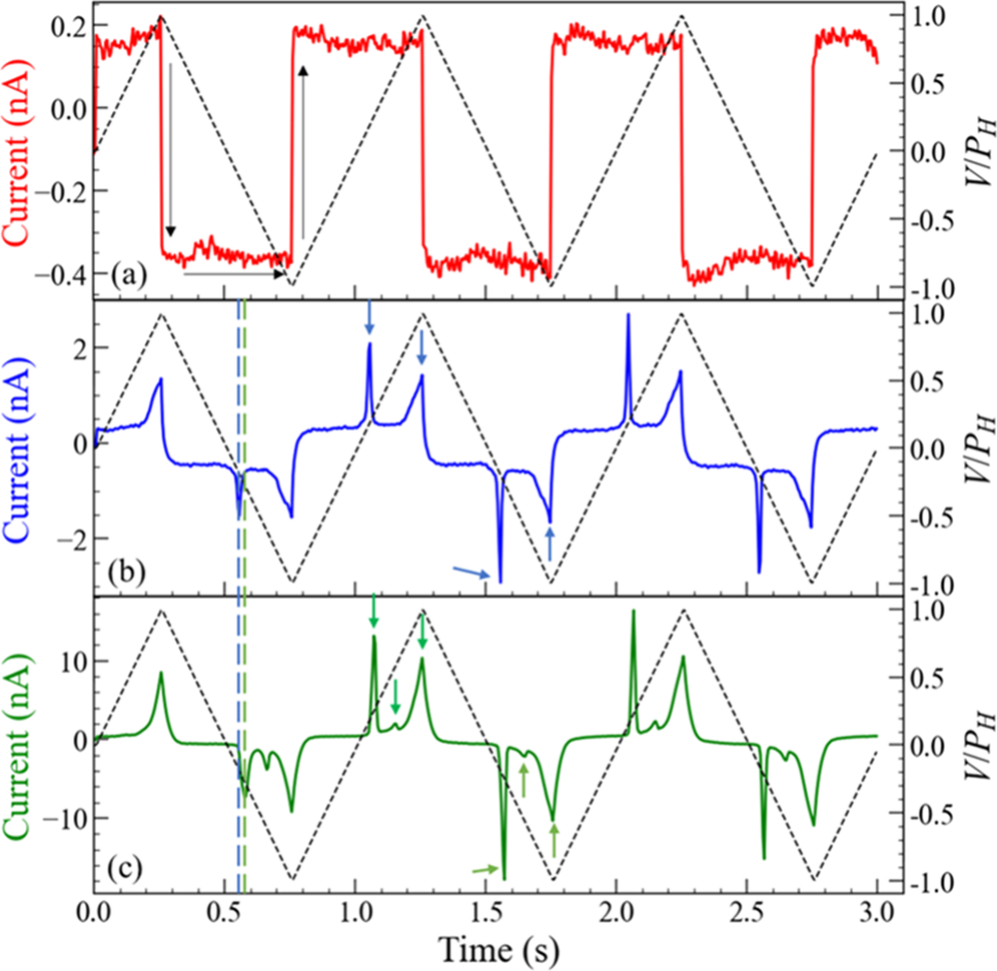
Three cycles of TVPs (right) with the pulse height (a) ±1.4 V, (b) ±2.0 V, and (c) ±2.6 V; and corresponding current waveforms (left). The pulse width was fixed at 500 ms. The voltage-pulse stream corresponds to “101010” because the positive and negative triangular voltage pulses represent “1” and “0” in the synthetic time-series data stream. The black arrows in panel (a) represent the direction of the rapid current waveform change. The dotted blue and green vertical lines in panels (b) and (c) compare the current peak positions in each current waveform. The blue and green arrows in panels (b) and (c) compare the number of current peaks for 1 cycle.

Figure 7a–c shows the time variation in the current values (*I*–*t* graphs) for TVP streams with the pulse height (*P*_H_) of 1.4, 2.0, and 2.6 V, respectively. The value of the pulse width (*P*_W_) was fixed at 500 ms. It should be noted that the vertical axes on the right in Figure 7 are the voltage values normalized by *P*_H_. As a control experiment to evaluate the noise from the measurement system, current values without the IL**-**reservoir were measured. The current noise level was sufficiently low to evaluate the electrical properties of the IL**-**reservoir (Supporting Information). At *P*_H_ = 1.4 V (Figure 7a), the sign of the current value switched from positive to negative (negative to positive) when the slope of the TVP switched from positive to negative (negative to positive). This is likely due to the charging and discharging of the electrical double layer (EDL) at the electrode/IL interface. The EDL-induced current is represented as *Q*/*t*, where *Q* is the charge accumulated at the EDL. Therefore, high-speed measurements (i.e., a small value of *t*) generally result in increased charging and discharging currents. However, in the IL-reservoir, the EDL current remained at approximately 10 and 2% of the total current at *P*_H_ = 2.0 and 2.6 V, respectively, and current peaks related to the Cu redox reactions can be clearly observed in Figure 7b,c. This is because the Pt-electrode area of the IL-reservoir is very small, thus decreasing the value of *Q*.^33^ When *P*_H_ increases from 2.0 to 2.6 V, the number of current peaks increases from 2 to 3, as indicated by the blue and green arrows in Figure 7b,c, respectively. For improved clarity, the current values for one cycle of TVP streams (current values for the time range from 1.0 to 2.0 s) are marked with arrows. In addition, the intensities of the current peaks for *P*_H_ = 2.6 V are higher than those for *P*_H_ = 2.0 V. Furthermore, as depicted by the blue and green vertical dotted lines in Figure 7b,c, the current peak shifted to the higher voltage side at *P*_H_ = 2.6 V compared to *P*_H_ = 2.0 V.

The current peak increase observed in Figure 7c indicates that the increased voltage caused the copper to react, along with other redox species including water and oxygen (Figure 6), although it is difficult to specify the redox reaction corresponding to each current peak. The current peak intensity increase at *P*_H_ = 2.6 V indicated that a large number of redox species, including Cu and Cu ions, participate in the redox reactions. The peak position shift could be attributed to the voltage-sweep rate difference between *P*_H_ = 2.6 and 2.0 V. Moreover, when *P*_W_ varied with a fixed *P*_H_, a similar current peak position shift was observed (see the Supporting Information). Also, *P*_W_ influences the information-processing performance, which is explained in the Supporting Information. In addition, the virtual-node number dependence of the information-processing performance is shown in the Supporting Information.

These results indicate that the number, intensity, and positions of the current peaks can be controlled by the voltage conditions, such as the voltage-pulse height and sweep rate.

### 3.4 Influence of the Voltage-Pulse Polarity Change

**Figure 8:**
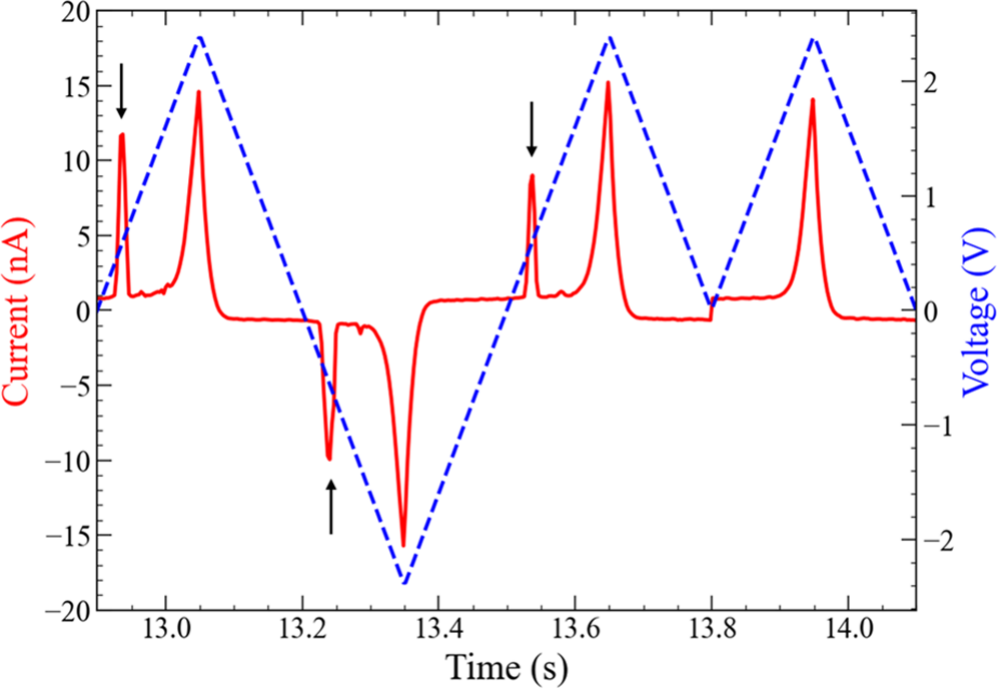
Input signal sequence-dependent current waveform (left) from the IL-reservoir device. The present voltage-pulse stream (right) corresponds to “1011”, which is part of the randomly arranged binary number sequence.

As indicated by the black arrows in Figure 8, the first current peaks in each time step are observed only when the voltage-pulse polarity changes. In contrast, the peaks are unobservable when voltage pulses with the same polarity are applied sequentially to the IL**-**reservoir. This current response can be attributed to the metal redox reactions. As discussed in the previous section, the reactions from Cu metal to Cu ions (oxidation) in one electrode and from Cu ions to Cu metal (reduction) in the opposite electrode generally occur simultaneously. However, when voltage pulses with the same polarity are applied sequentially, the Cu metal on one of the electrodes is exhausted by the preceding pulse voltage; hence, further redox reactions are inhibited by the sequential input of same polarity voltage pulses. Therefore, this intrinsic relationship between the faradaic current peak and voltage polarity change results in a noticeable difference in the current waveform, depending on the sequence of 1 and 0 in the synthetic time-series signal. The influence of experimental conditions, including the measurement cycle, temperature, and pulse voltage width, on the faradaic current peak is shown in the Supporting Information. In addition, the influence of the device structure, including the electrode distance and electrode area, is briefly explained in the Supporting Information.

### 3.5 Time-Series Data Processing

**Figure 9:**
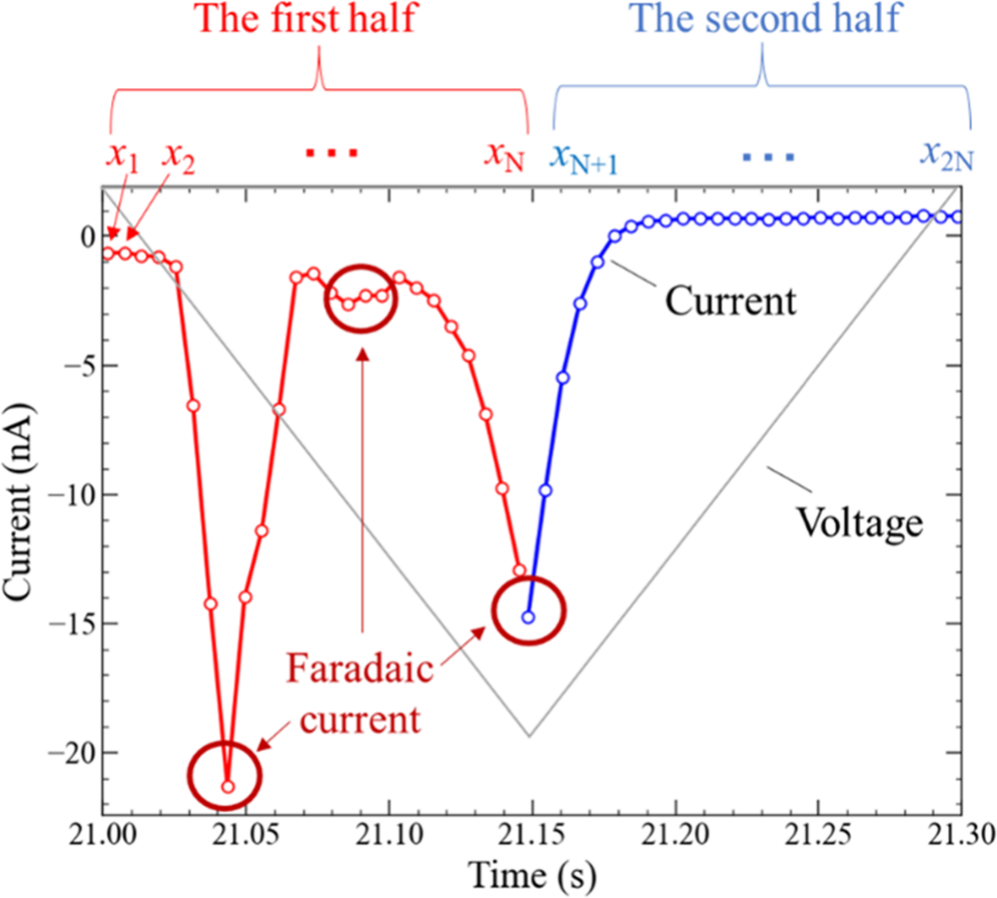
Splitting of the current waveform (red and blue open circles) observed under the negative TVP (gray solid line) into two parts: first and second half. The current value in the first half is denoted as *x_i_* (*i* = 1, 2, ..., *N*) and is the input to the *i*th virtual node. The current value in the second half is denoted as *x_j_* (*j* = *N* + 1, *N* + 2, ..., 2*N*) and is the input to the *j*th virtual node. As depicted by the red circles, the faradaic current peaks are observed mostly in the first half.

In this section, the impact of faradaic current on the calculation performance of a physical reservoir will be discussed. The data processed by the IL**-**reservoir were virtual time-series binary data consisting of randomly aligned 1 and 0. The 1 and 0 values were replaced with positive and negative TVPs, respectively, as described in Section 2. The STM task was used to evaluate short-term memory characteristics, whereas the PC task was used to evaluate the nonlinear transformation performance of the input signal. To investigate the importance of the faradaic current for these tasks, two experimental conditions were tested (Exp-1 and Exp-2), where Exp-1 involved the virtual-node selection method. The virtual nodes were divided into two parts: the first and second halves, as shown in Figure 9. The current peak corresponding to the faradaic current appeared more dominantly in the first half, and the calculation performance was compared using the current values of these two parts separately. The datasets for the first and second parts were defined as dataset-F and dataset-L, respectively. Furthermore, as a control experiment, the calculation performance using all of the virtual nodes was evaluated, and the dataset for this calculation condition was named dataset-A.

**Figure 10:**
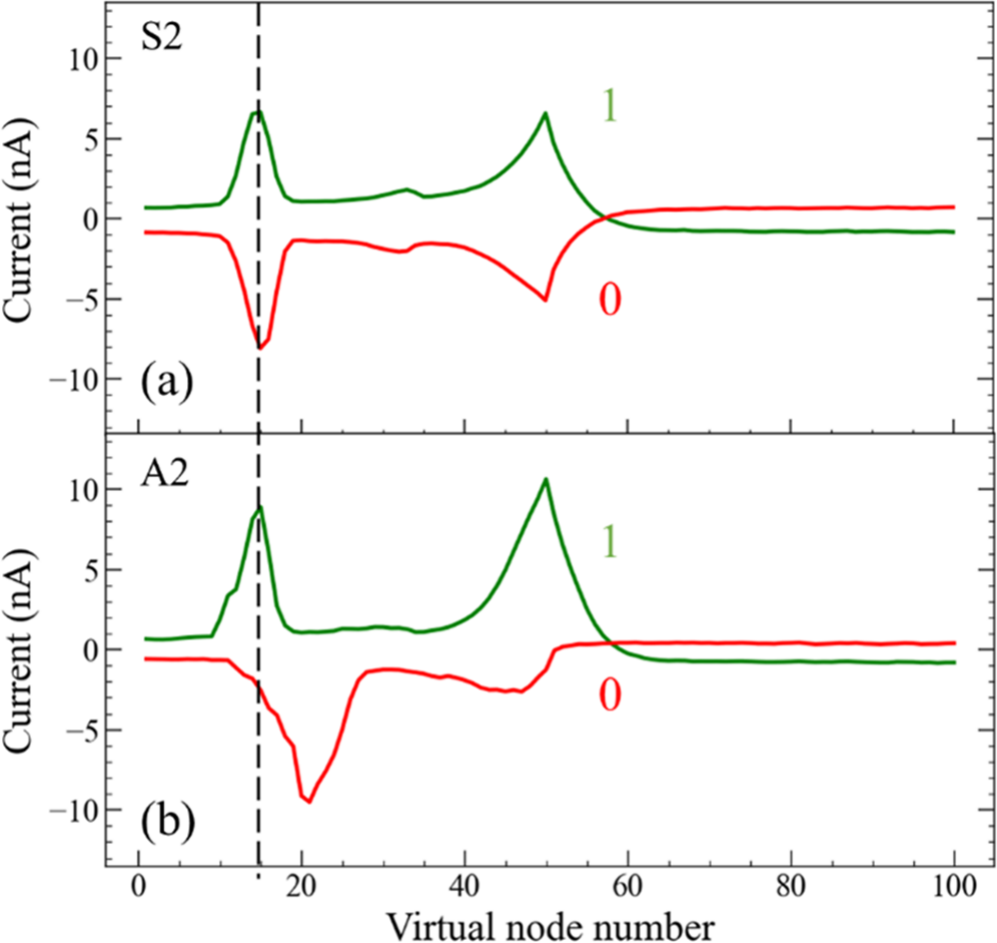
Current waveform for 1 (green) and 0 (red) under the measurement conditions of (a) S2 and (b) A2 as a function of the virtual node number when the TVP “10101010...” was applied to the IL-reservoir device.

Exp-2 is related to the symmetry of the TVP height, as summarized in Table 1. Hereafter, the *P*_H_ values for the positive and negative voltage pulses are denoted as *V*_P_ and *V*_N_, respectively. For measurement conditions S1 and S2, the *P*_H_ values for 1 and 0 were identical. For instance, in the case of S2, *V*_P_ and *V*_N_ are +2.4 and −2.4 V, respectively. For measurement conditions A1 and A2, the value of *P*_H_ for 0 is different from that for 1. For instance, in the case of A2, *V*_P_ and *V*_N_ are +2.4 and −1.6 V, respectively. Figure 10a shows the current values for data 1 and 0 obtained under condition S2 plotted as a function of the virtual node number. On the other hand, Figure 10b shows the current values for data 1 and 0 under condition A2. The current waveforms for data 1 in S2 and A2 were nearly identical. In addition, for S2, the current waveforms for data 0 exhibited a line-symmetric shape with reference to the horizontal axis compared with those for data 1. The most distinctive feature of the current waveform was observed for data 0 in A2. As shown by the dotted vertical lines in Figure 10a,b, the peak position for the faradaic current for data 0 in A2 shifts toward a higher number of virtual nodes. Simultaneously, the current values at a virtual node number of ∼50 significantly decreased for data 0 in A2 compared with the other three cases. These differences in the current waveforms improved PRC performance under the A2 condition compared to S2.

**Table 1:** Comparison of the Four Measurement Conditions (S1, S2, A1, and A2) to Investigate the Role of the Voltage-Pulse Symmetry^a^

measurement condition name	symmetry of voltage pulse for input data 1 and 0	*V*_P_ (V)	*V*_N_ (V)	*P*_W_ (ms)
S1	symmetric	+1.6	–1.6	300
S2	symmetric	+2.4	–2.4	300
A1	asymmetric	+2.4	–2.0	300
A2	asymmetric	+2.4	–1.6	300

a *V*_P_ and *V*_N_ are the voltage-pulse heights for the input data 1 and 0, respectively. The voltage pulses for 1 and 0 are symmetric under conditions S1 and S2 (i.e., |*V*_N_| = *V*_P_). They are also asymmetric under conditions A1 and A2 (i.e., |*V*_N_| < *V*_P_). The pulse width (*P*_W_) was fixed at 300 ms.

**Figure 11:**
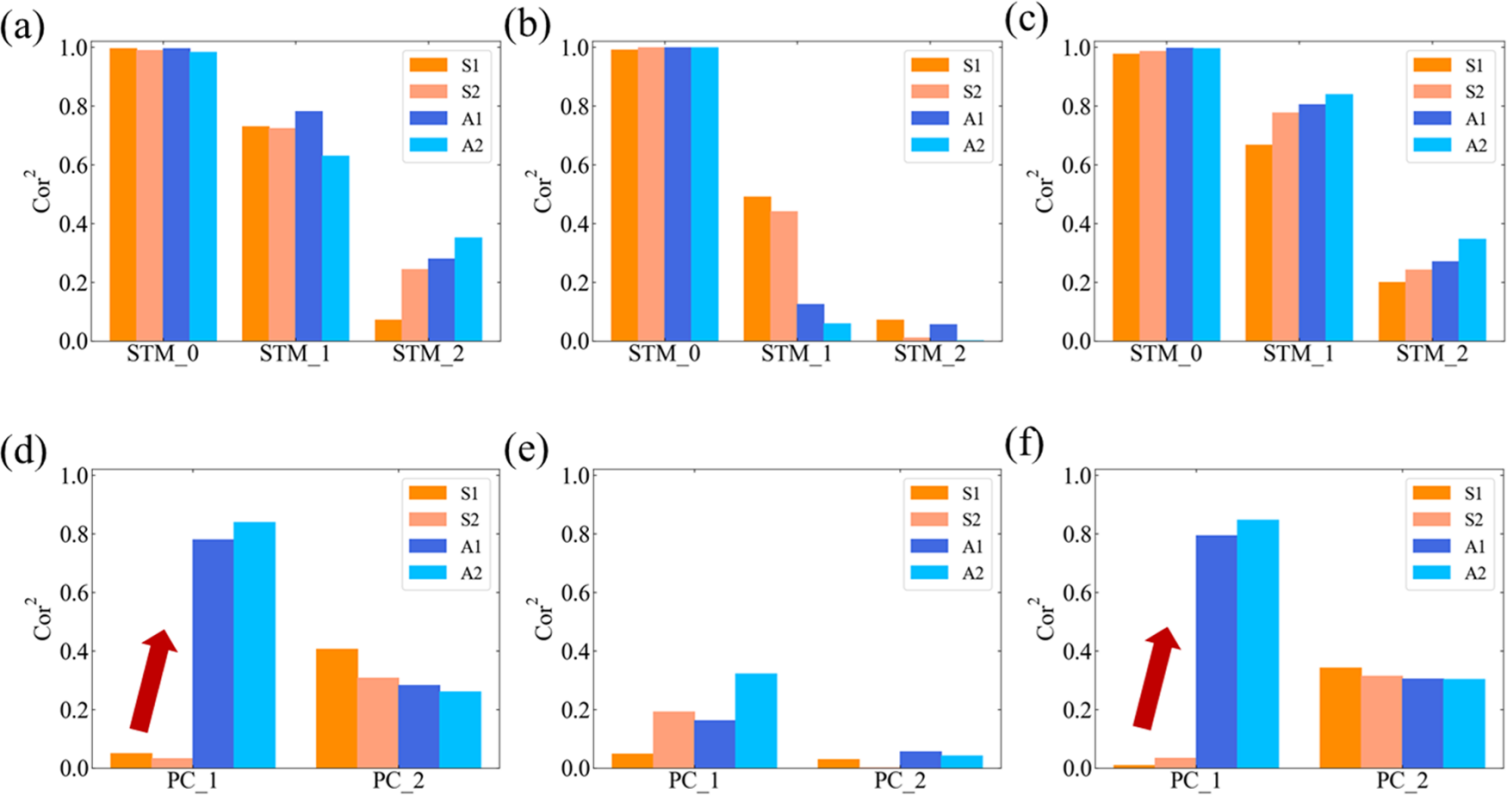
Squared value of the correlation coefficients (Cor^2^) for (a)–(c) STM and (d)–(f) PC tasks with various *T*_delay_ values. *T*_delay_ = *i* for the STM_*i* task (*i* = 0, 1, and 2) and *T*_delay_ = *j* for the PC_*j* task (*j* = 1 and 2). The datasets used for those tasks are (a, d) dataset-F, (b, e) dataset-L, and (c, f) dataset-A. The colored bars correspond to the four measurement conditions described in Table 1 (S1: orange, S2: light orange, A1: blue, and A2: light blue). The red arrows in panels (d) and (f) indicate the remarkable Cor^2^ increase observed when the measurement conditions were changed from symmetric (S1 and S2) to asymmetric (A1 and A2) voltage conditions.

Figure 11a–c shows the square of the correlation coefficient evaluated for the STM tasks when using datasets F, L, and A, respectively. Four colors in the bar charts correspond to the four different measurement conditions listed in Table 1 (S1, S2, A1, and A2). Similarly, the square of the correlation coefficient values evaluated for the PC tasks is depicted in Figure 11d–f. In the present study, each of STM and PC tasks was executed three times and the averaged value of the correlation coefficient was used to draw the bar charts in Figure 11a–f. The details on the evaluated correlation coefficient values used to calculate the average values for Figure 11a–f are summarized in Tables S2 and S3. Hereafter, the square value of the correlation coefficient for task X using dataset-Y is represented as Cor^2^(X, Y), where
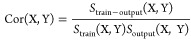
3Here, *S*_train_(X, Y) and *S*_output_(X, Y) are the variances of the training and output data corresponding to task X using dataset-Y. In contrast, *S*_train-output_(X, Y) is the covariance between the training and output data. For example, Cor^2^(STM_1, F) and Cor^2^(STM_1, L) denote the square values of the correlation coefficient for the STM_1 task using datasets-F and -L, respectively. The training and output data used to calculate Cor^2^(STM_1, F) and Cor^2^(STM_1, L) are provided in the Supporting Information.

First, the influence of virtual node selection (Exp-1) on STM tasks was evaluated. For example, in S2, the values of Cor^2^(STM_i, F)/Cor^2^(STM_i, A) were determined to be 1.00, 0.93, and 1.00 for *i* = 0, 1, and 2, respectively, while the Cor^2^(STM_i, L)/Cor^2^(STM_i, A) ratios were 1.01, 0.56, and 0.04, respectively. For *i* = 0, the differences between Cor^2^(STM_0, F), Cor^2^(STM_0, L), and Cor^2^(STM_0, A) were negligible. In contrast, for *i* = 1 and 2, the values of Cor^2^(STM_i, L) were much smaller than those of Cor^2^(STM_i, F) and Cor^2^(STM_i, A). A similar trend was also observed for S1, A1, and A2, as shown in Figure 11a–c. These results clearly show that STM accuracy can be improved with dataset-F. Therefore, it is evident that the faradaic current plays a role in improving the short-term memory characteristics of the IL**-**reservoirs. It should be noted that the abovementioned impact of the faradaic current was independent of the weight update method, indicating that this represented an intrinsic property of the IL**-**reservoir (see the Supporting Information for more details).

For PC tasks, both the virtual node selection and symmetry of the input signal influenced the calculation performance. For the PC_1 task using dataset-F, the values of Cor^2^(PC_1, F) for A1 and A2 were at least 15 times larger than those for S1 and S2 when the nearest values were compared, highlighting the advantage of using faradaic currents to improve the nonlinear transfer performance of the IL**-**reservoir. This trend was noticeably applicable to Cor^2^(PC_1, A), whereas the impact of input signal asymmetrization on the PC_1 task accuracy was very small for dataset-L, as shown in Figure 11e. We used t-distributed stochastic neighbor embedding (t-SNE) to examine the changes in the accuracy of STM and PC tasks.

### 3.6 t-Distributed Stochastic Neighbor Embedding (t-SNE)

**Figure 12:**
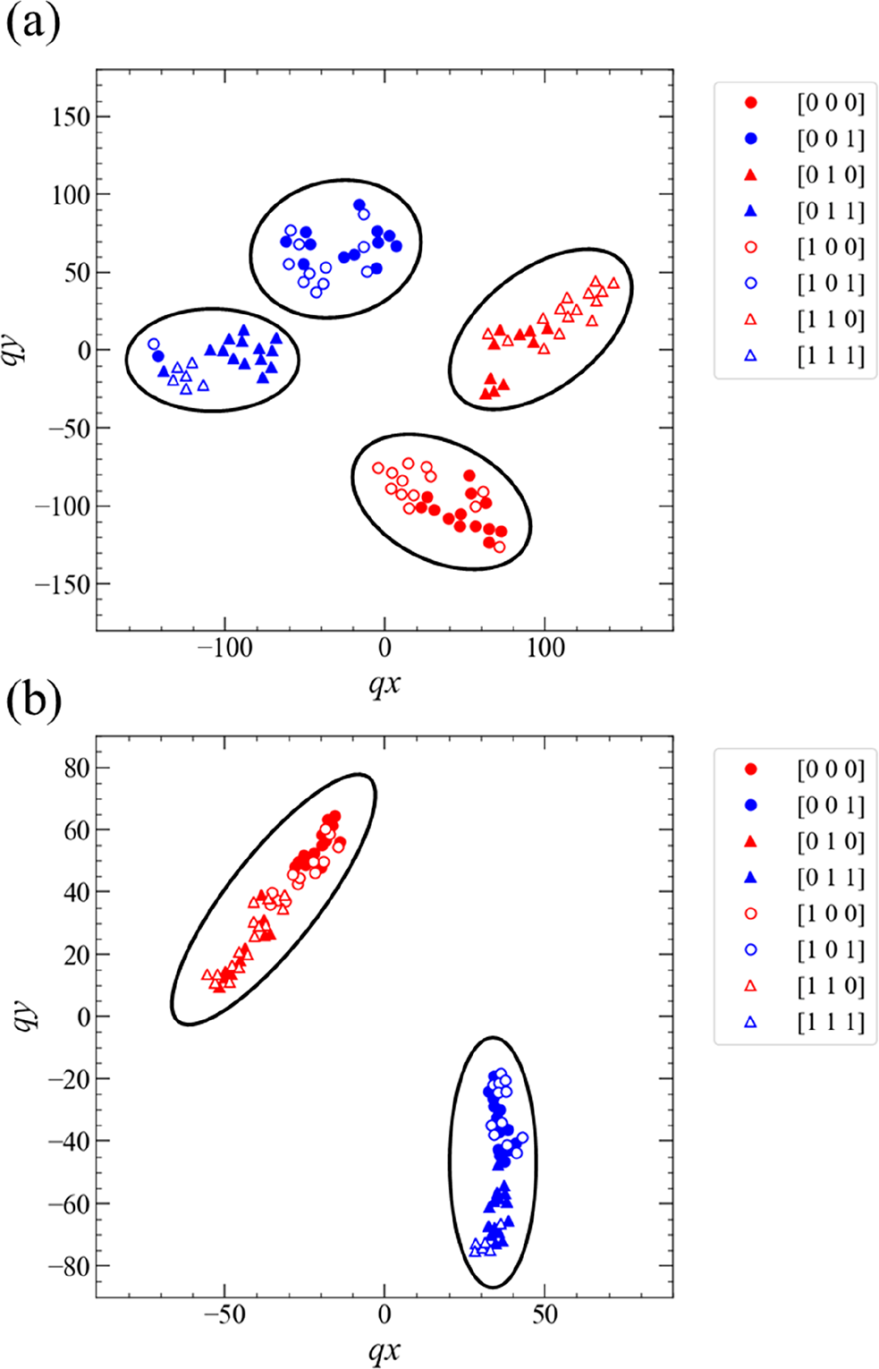
Visualization of the STM_2 task using t-SNE when (a) dataset-F and (b) dataset-L were used as the original high-dimensional data. The blue and red colors correspond to 1 and 0 in time step *T*. The triangular and circular symbols correspond to 1 and 0 in time step *T*-1. The open and solid symbols correspond to 1 and 0 in time step *T*-2.

The calculation algorithm known as t-SNE compresses high-dimensional data into two-dimensional (2D) space, allowing high-dimensional data to be represented visually.^34,35^ It was previously shown that this method can be applied to STM and PC.^4^ Therefore, t-SNE was applied to the dataset used for the STM and PC tasks and the STM task results are shown in Figure 12. Here, dataset-F in S2 (Figure 12a) and dataset-L in S2 (Figure 12b) were used for the t-SNE analysis. The data label in Figure 12 was determined by the numerical sequence of the binary data (1 and 0) in a continuous sequence of three time steps (*T*-2, *T*-1, and *T*). The blue and red colors correspond to 1 and 0 in time step *T*. The triangular and circular symbols correspond to 1 and 0 at time step *T*-1. The open and solid symbols correspond to 1 and 0 in time step *T*-2. Based on these definitions, the blue circular solid symbols represent the numerical sequence “001”. As represented by the black circles in Figure 12a, dataset-F was classified into four groups depending on the symbol color and shape. However, for solid and open symbols with the same symbol shape, further grouping became difficult because these symbols are mixed, except for the triangular symbols. These results indicate the following characteristics of dataset-F: data in time step *T* clearly remember the information contained in time step *T*-1, while mostly forgetting the information from time step *T*-2. As shown in Figure 12b, dataset-L provides different t-SNE analysis results compared to those of dataset-F. Although dataset-L can be classified into two groups depending on the symbol color, it was difficult to further classify based on the symbol shape. Therefore, in the case of dataset-L, data in time step *T* lost most of the memory, even for information in time step *T*-1. The t-SNE analysis results are consistent with the dataset-dependent calculation accuracies for the STM tasks, as shown in Figure 11a,b. It should be noted that Cor^2^(STM_1, F) is at least one and a half times larger than Cor^2^(STM_1, L) for the STM_1 task compared to the nearest value condition (S1). Furthermore, Cor^2^(STM_2, F) is less than half of Cor^2^(STM_1, F) compared to the nearest value condition (A2).

**Figure 13:**
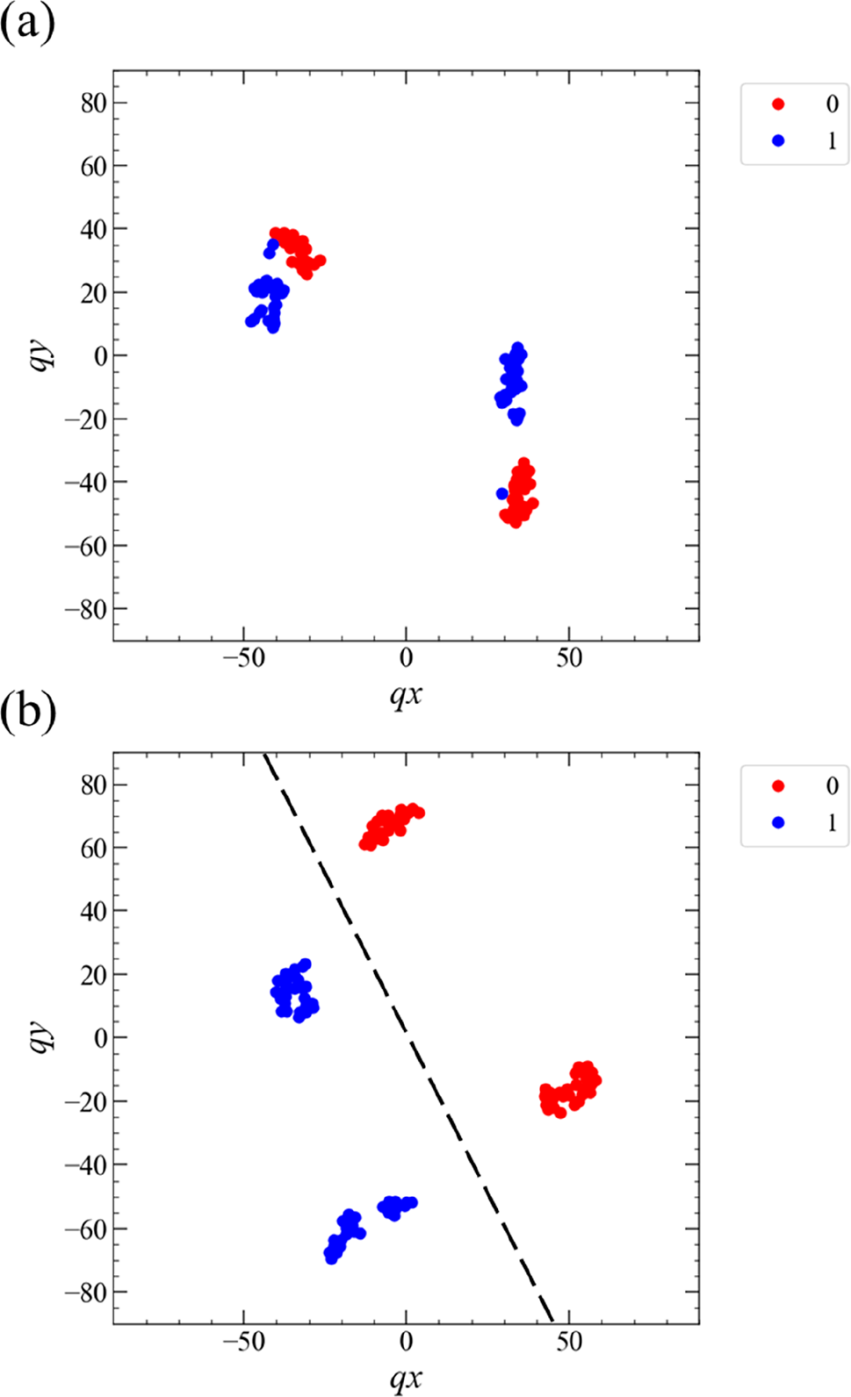
Visualization of the PC_1 task using t-SNE when the measurement conditions (a) S2 and (b) A1 were applied. The dataset used as the original high-dimensional data in panels (a) and (b) was dataset-A in both cases. The blue and red solid circles correspond to the target data 1 and 0 for the PC_1 task. The dotted line in panel (b) was drawn based on linear regression using the dimension-reduced dataset-A by the t-SNE algorithm.

The t-SNE analysis results for the PC_1 task are shown in Figure 13. Dataset-A in S2 (Figure 13a) and A1 (Figure 13b) were used as data for the t-SNE analysis. For S2, the two-dimensional (2D) data represented by the blue and red circles after the dimensional reduction by the t-SNE algorithm are plotted in a point-symmetric fashion to the origin of the *qx*–*qy* plane in Figure 13a. Consequently, dividing this 2D space into two subspaces for the blue and red circles with a straight line is difficult. However, for A1, the 2D data represented by the blue and red circles can be separated into two subspaces with a straight line (Figure 13b), which was determined by the linear regression of the 2D data. These results indicate that the linear separability of dataset-A obtained under condition A1 for the PC_1 task was much higher than that obtained under condition S2, even in the original high-dimensional space.

In Table 2, the operating mechanism and performance (e.g., pulse width, switching voltage, output current value, short-term memory (STM), and parity-check (PC) task accuracy) for previously reported physical reservoir devices were compared with those of the newly developed IL**-**reservoir.^4,5,13^ Compared to other physical systems, low power consumption in the novel IL**-**reservoir can be expected because the output current value is very small. The timescale of the device operation in the IL**-**reservoir developed herein is several hundred milliseconds, analogous to the timescale of biological reactions. Therefore, the IL**-**reservoir described herein is appropriate for processing time-series data generated from biological reactions. Because this IL-reservoir and polyoxometalate molecule (PM) devices have similar operating mechanisms (electrochemical reaction), some operating performances, including the switching voltage become comparable. However, a much lower power device operation compared to the PM device was achieved by introducing a microelectrode structure, which is suitable for time-series data processing in the edge region.

**Table 2:** Comparison of the Operating Mechanism and Performance of Select Physical Reservoir Devices Reported Previously and the IL-Reservoir Developed Herein

structure	FeFET device	spintronics device	polyoxometalate molecule device	IL device (this study)
mechanism	ferroelectricity	magnetic tunnel junction	electrochemical reaction	electrochemical reaction
switching voltage range	0.5–3 V	44 mV	2 V	1.6–3.6 V
power (current)	∼100 μA	∼100 μA	∼100 μA	10–20 nA
pulse width range	2–200 μs	10–100 ns	2–10 ms	0.5–900 ms
reliability performance				900
MC (STM)	2.1 (*T*_delay_ = 1–9)	2.3 (*T*_delay_ = 1–30)		1.1 (*T*_delay_ = 1–2)
MC (PC)	2.0 (*T*_delay_ = 1–9)	2.4 (*T*_delay_ = 1–30)		1.1 (*T*_delay_ = 1–2)
remark	ref (4)	ref (5)	ref (13)	

## 4 Conclusions

A physical RD was successfully prepared based on an IL**-**reservoir, exhibiting high sensitivity for detecting faradaic current at the metal-ion IL/electrode interface under fast voltage-pulse conditions. This sensitivity was achieved by reducing the effective electrode area to the submicrometer scale. RD performance was evaluated by applying synthetic time-series data consisting of binary (1 and 0) sequences in the form of TVPs. The faradaic current peaks improved the short-term memory characteristics of the IL**-**reservoir. Moreover, the current signals generated as TVPs with different voltage levels for 1 and 0 improved the nonlinear transformation performance of the IL**-**reservoir. These advantageous effects of the faradaic current are well explained by two-dimensional data mapping based on t-SNE.

## Supporting Information Available

Operando observation method; Cu-G3 during XPS measurements; peak origin at Raman spectroscopy; CV measurement in vacuum; and pulse width dependence as well as output data and training data for the STM_1 task (PDF)
