# Supplementary material for: Dynamic Nonlinear Behavior of Ionic Liquid-Based Reservoir Computing Devices
Source: ACS Appl Mater Interfaces. 2022 Jul 26;14(32):36890–901. doi: 10.1021/acsami.2c04167 (PMC9389526; doi:10.1021/acsami.2c04167)
Supplement: Supplementary file 1 — am2c04167_si_001.pdf [file am2c04167_si_001.pdf]

Supporting Information

Dynamic Nonlinear Behavior of Ionic Liquid-Based Reservoir Computing Devices

Takuma Matsuo[1,2], Dan Sato[1,2], Sang-Gyu Koh[1,2], Hisashi Shima[2,*],

Yasuhisa Naitoh[2], Hiroyuki Akinaga[2], Toshiyuki Itoh[3],

Toshiki Nokami[4], Masakazu Kobayashi[5], and Kentaro Kinoshita[1,*]

1 Department of Applied Physics, Graduate School of Science, Tokyo University of Science, Katsushika, Tokyo 125-8585, Japan

2 Device Technology Research Institute, National Institute of Advanced Industrial Science and Technology, Tsukuba, Ibaraki 305-8565, Japan

3 Toyota Physical and Chemical Research Institute, Nagakute, Aichi 480-1192, Japan

4 Center for Research on Green Sustainable Chemistry, Faculty of Engineering, Tottori University, Koyama, Tottori 680-8552, Japan

5 New Value Creation Office, NAGASE & CO., LTD., Nihonbashi, Tokyo 103-8355, Japan

*Corresponding author: Kentaro Kinoshita (e-mail: kkinosita@rs.tus.ac.jp) and Hisashi Shima (e-mail: shima-hisashi@aist.go.jp)

CONTENTS

**Figure S1**. The photograph of the experimental setup for operand observation of IL-reservoir operation.	----	S3
**Figure S2.** Experimental Raman spectrum and calculated Raman activity as a function of the Raman shift.	----	S5
**Table S1.** Origin of Raman peaks estimated from the quantum-chemical calculation.	----	S6
**Figure S3.** The photograph of the Cu-G3 droplet in air before the XPS measurement.	----	S7
**Figure S4.** CV characteristics in vacuum and optical microscope image.	----	S8
**Figure S5.** Pulse width dependency when triangular voltage pulse is applied.	----	S9
**Figure S6.** Training and output data for STM_1 task as a function of timestep *T*.	----	S10
**Figure S7.** XPS survey spectrum for Cu-G3.	----	S11
**Figure S8.** *I-V* profiles of three IL-reservoirs under different voltage conditions.	----	S13
**Figure S9.** The optical microscopy observation results in Devices S1, S2, and S3 immediately, after 5 min and after 25 min.	----	S13
**Figure S10.** Three input TVPs with the wait time T_{wait} introduced between 2nd and 3rd TVP.	----	S14
**Figure S11.** T_{wait} dependence of the *I-V* curve.	----	S15
**Figure S12.** The time variation in current measured without or with the IL-reservoir.	----	S16
**Figure S13.** Measurement condition (pulse width and temperature) dependence of the *I-V* curve.	----	S19
**Figure S14.** Temperature and pulse width dependence of cumulative probability of the Faradaic current value.	----	S19
**Figure S15.** The influence from voltage pulse width of input TVPs and virtual node number on the information processing accuracy for the STM tasks.	----	S20
**Figure S16.** Optical microscope images for the IL-reservoirs to evaluate the influence of the interelectrode distance.	----	S22

**Figure S17.** Inter-electrode distance dependence of the *I-V* curves.	----	S23
**Figure S18.** Optical microscope of the device top view and corresponding schematic of the cross-section for the IL-reservoir prepared to evaluate the influence of the electrode area.	----	S24
**Figure S19.** Electrode area dependence of *I-V* curve measured using the IL-reservoir shown in Figure S18.	----	S25
**Figure S20.** Electrode area dependence of the STM task accuracy.	----	S26
**Figure S21.** Weight update method dependence of the STM task accuracy.	----	S27
**Table S2,3.** The detail on the evaluated correlation coefficient values (Cor) for the STM and PC tasks, respectively.	----	S28

Shown in Figure S1(a) is the photograph of the experimental setup for operand observation of IL-reservoir operation, which accompanies Cu deposition and dissolution on the Pt electrodes. Since the spacing between the objective lens and the sample stage of the optical microscope is limited, we used a breadboard, the dedicated BNC terminals, and CDIP (Ceramic Dual Inline Package) shown in Figure S1(b) to measure the *I-V* characteristics of IL-reservoir under the objective lens. As shown in Figure S1(c), the electrodes of the IL-reservoir were wire-bonded to the electrical pads on the CDIP.

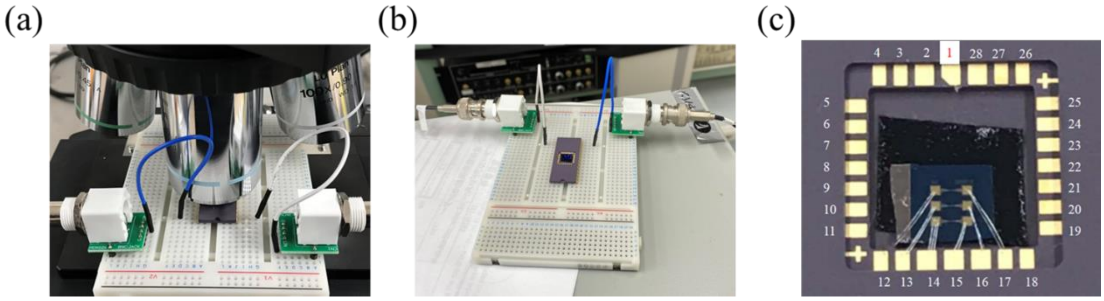

Figure S1. (a) Photograph of the experimental setup for operand observation of IL-reservoir operation, (b) breadboard, the dedicated BNC terminals, and CDIP used for the present operand observation, and (c) IL-reservoir mounted on the CDIP.

In order to conduct the peak assignment for the experimental Raman peaks, the quantum chemical calculation was carried out. From the calculated results, 8 peaks (peak A, C, D, E, F, G, J and L in Figures S2(a) and (b)) were assigned to the optimized molecular structure of Cu-G3 in Fig. 4(d). 6 peaks (peak B, G, H, I, K and M in Figures S2 (a) and (c)) were assigned to the optimized molecular structure of Cu-G3 in Fig. 4(e). The detail of the peak origin for those 13 peaks were summarized in Supplementary Table 1. It should be noted that, different from the experimental Raman spectroscopy in Figures S2(a), the Raman activity was plotted in Figures S2(b) and Figures S2(c), which is unproportional to the experimental Raman peak intensity. Therefore, some of the calculated Raman activity becomes quite low even if there are corresponding experimental Raman peaks. The slight difference between the peak positions in Figures S2(a), (b) and (c) are attributed to the intermolecular interaction in the Cu-G3 used in the Raman spectroscopy. Namely, the calculation was carried out for the single pair of $Cu(Tf_2N)_2$ and G3, while a collection of those molecules was measured in the experimental Raman spectroscopy.

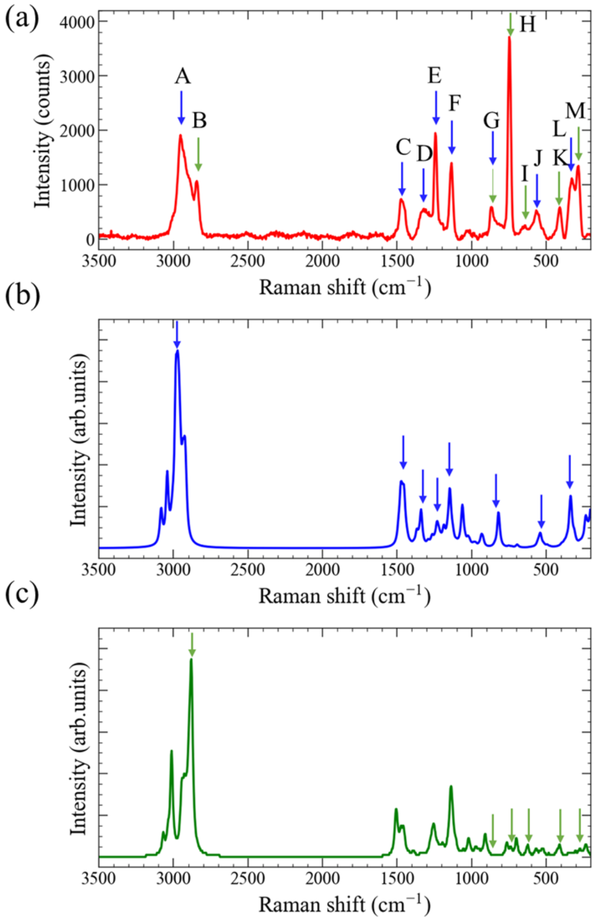

Figure S2. (a) experimental Raman spectrum and (b), (c) calculated Raman activity as a function of the Raman shift. All the peaks (peak A to M) are inherent in the optimized molecular structure of Cu-G3 in Fig. 4(d) and Fig. 4(e). The origin of those peaks is summarized in Table S1.

Table S1. Origin of Raman peaks estimated from the quantum-chemical calculation

Peak	Raman shift in experiment(cm-1)	Raman shift in calculation (cm-1)	Estimated origin	molecular structure
A	2950	2984	C-H stretching vibration in G3	MC1
B	2850	2881	C-H stretching vibration in G3	MC2
C	1470	1475	C-H bending vibration in G3	MC1
D	1320	1338	C-H bending vibration in G3	MC1
E	1240	1228	C-H and C-O bending vibration in G3, S-O stretching vibration in Tf_2N	MC1
F	1130	1146	C-H and C-O bending vibration in G3	MC1
G	870	889	C-O and Cu-O stretching vibration in G3, C-O bending vibration in G3	MC2
		844	C-O and Cu-O stretching vibration in G3, C-O bending vibration in G3	MC1
H	740	736	S-C stretching vibration in Tf_2N, C-F, S-O and S-N bending vibration in Tf_2N	MC2
I	640	625	Cu-O, C-F, S-O and S-N bending vibration in Tf_2N	MC2
J	560	539	C-O bending vibration in G3, C-F and S-O bending vibration in Tf_2N	MC1
K	400	410	Cu-O stretching vibration in Tf_2N, C-F, S-O and S-N bending vibration in Tf_2N	MC2
L	330	334	Cu-O stretching vibration between Cu and G3, C-O bending vibration in G3, S-N bending vibration in Tf_2N	MC1
M	280	274	Cu-O, S-N, S-O and C-F bending vibration in Tf_2N	MC2

Shown in Figures S3(a) – (c) are the photograph of the Cu-G3 (the solvated IL, $Cu(Tf_2N)_2$: Triglyme (G3) = 1 : 1) droplet in air before the XPS measurement, in vacuum during the XPS measurement, and in the re-exposure state to the air after the XPS measurement, respectively. The appearance change in Figures S3(b) is reasonably attributed to the evaporation of water in Cu-G3 in vacuum because present Cu-G3 involves relatively large amount of water according to the Karl Fischer titration result. On the other hands, the appearance change in Figures S3(c) is thought to be caused by the moisture absorption from the air.

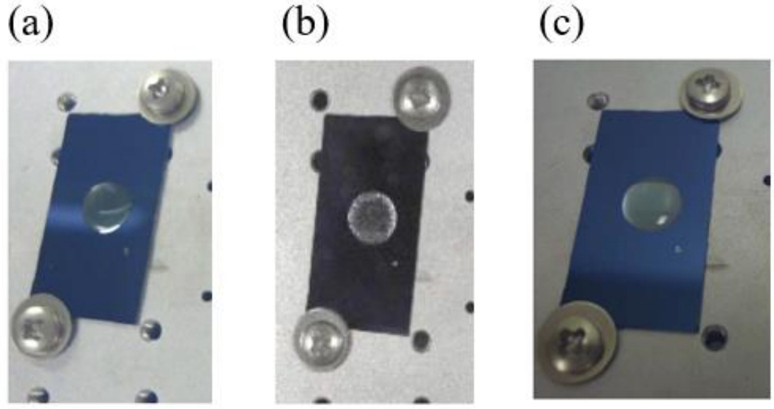

Figure S3. Photograph of the Cu-G3 droplet on the SiO_2/Si substrate (a) in air before the XPS measurement, (b) in vacuum during the XPS measurement, and (c) in the re-exposure state to the air after the XPS measurement.

We measured *I-V* characteristics in vacuum to investigate Cu deposition mechanism. Figures S4(a) is the CV curve measured using the IL-reservoir in the as-fabricated state in vacuum. Figure S4 (c) is the optical microscope image of the IL reservoir before the CV measurement in Figure 4(a). The output current was quite small and no appearance change of the IL-reservoir was observed. On the other hand, Figure S4 (b) is the CV curve measured in vacuum after the 2 cycles of the CV measurement in air. Figure S4 (d) is the optical microscope image of the IL-reservoir taken immediately after the above-mentioned CV curve measurement in air. In this case, Faradaic current was markedly observed, different from Figure S4(a). This result indicates that redox reaction in the first sweep requires the contribution of the redox species such as H_2O and O_2 in air rather than the decomposition of IL.

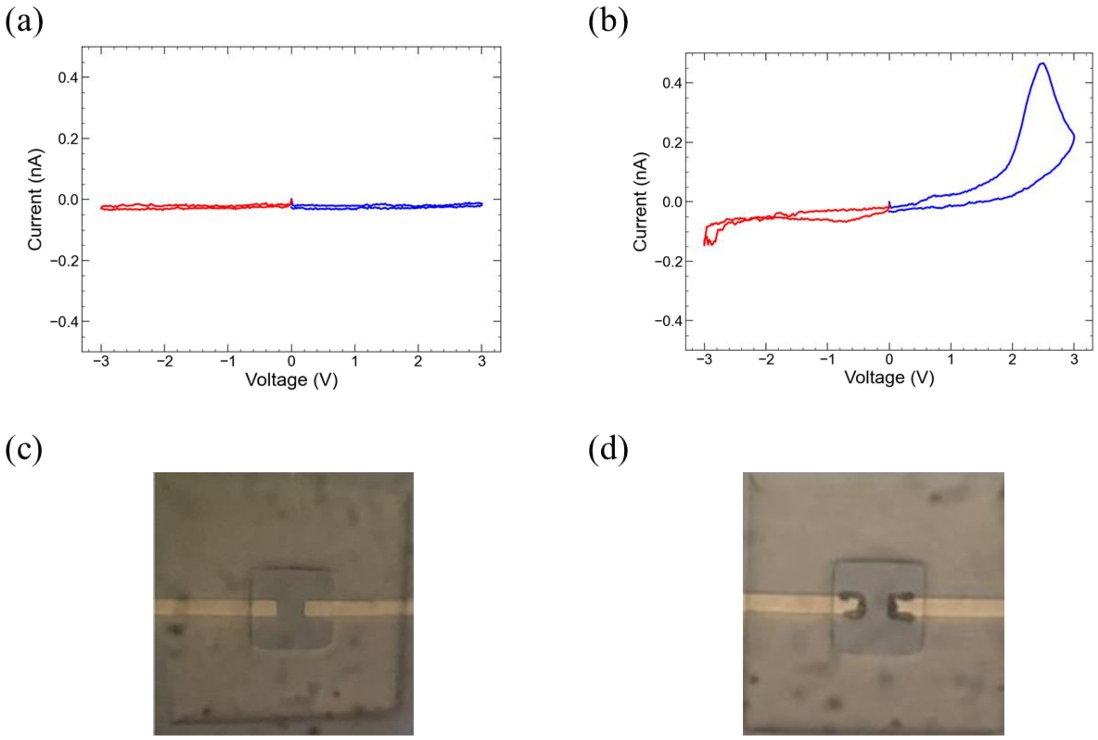

Figure S4. *I-V* characteristic (a) in vacuum without Cu deposition (b) in vacuum after depositing Cu. (c) optical microscope images before applying voltage in (a). (d) is optical microscope images before applying voltage in (b).

We investigated the influence of the voltage pulse width on the Faradaic current peak position. Shown in Figure S5(a) is the output current for the triangular voltage pulse streams with the pulse width of 100, 300, 500, and 700 ms. The horizontal axis in Figure S5(a) is normalized by the pulse width. As indicated by the vertical dotted lines, the Faradaic current peak shifted toward the higher voltage side with decreasing the voltage pulse width. Relationship between the voltage pulse width and the voltage value corresponding to the Faradaic current peak position was plotted in Figure S5 (b). The Faradaic current peak position shift toward the higher voltage side by decreasing the voltage pulse width observed in the wide range of the voltage pulse width condition from 100 to 10000 ms.

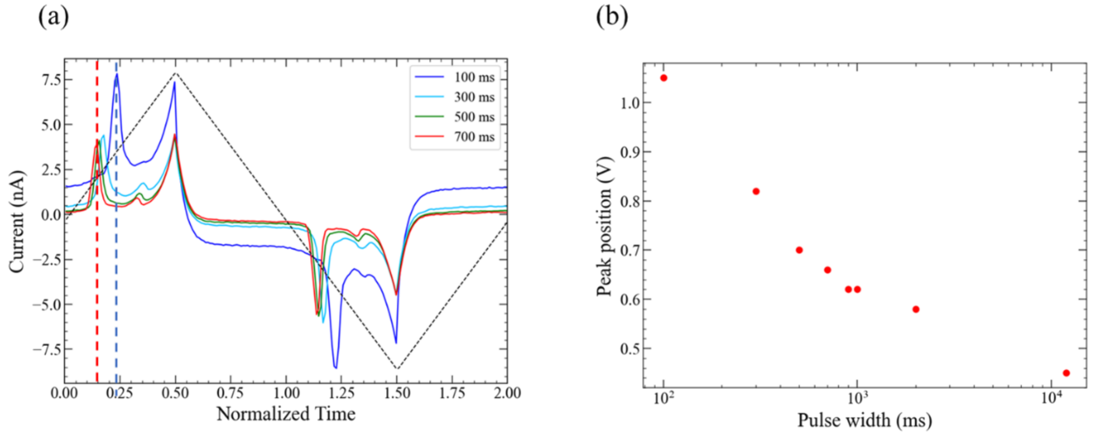

Figure S5. (a) Output current for the triangular voltage pulse streams with the pulse width of 100, 300, 500, and 700 ms, (b) the relationship between the voltage pulse width and the voltage value corresponding to the Faradaic current peak position.

Synthetic time-series data consisting of randomly arranged binary number (1 and 0) sequences input into IL-reservoir. We represent the STM tasks with $T_{\mathrm{delay}} = i$ as STM_i. As shown in Figure S6(a), the output data coincided well with the training data for the STM_1 task when dataset-F was used. On the other hand, as shown in Figure S6(b), the difference between the output data and training data was large when dataset-L was used. As a result, Cor^2(STM_1, F) becomes larger than Cor^2(STM_1, L).

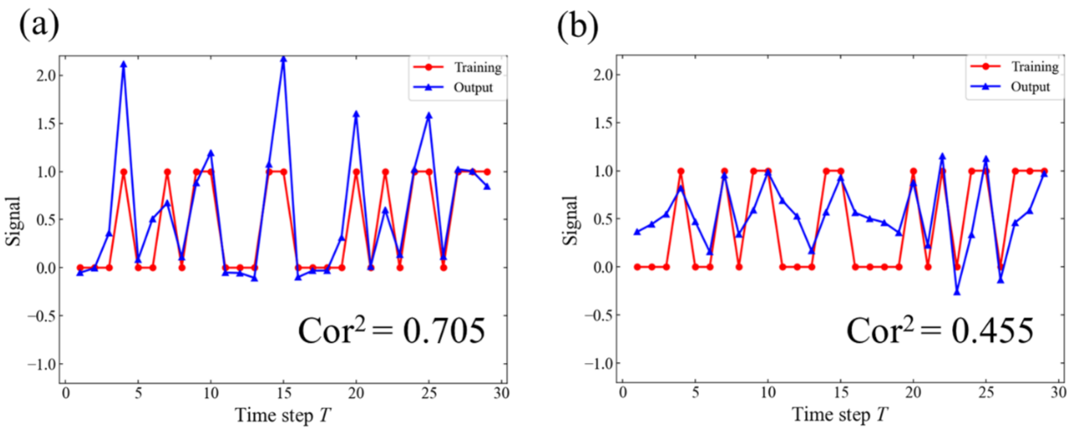

Figure S6. Training and output data for STM_1 task using (a) dataset-F and (b) dataset-L in condition S2 as a function of timestep T.

Figure S7 is the survey spectrum for Cu-G3. The almost all the detected elements can be attributed to either $Cu(Tf_2N)_2$ or G3 except for the faint signal of Si. The Si 2s and Si 2p signals are originated from the minute amount of the Si wafer fragment, which is generated when the Si wafer is cleaved.

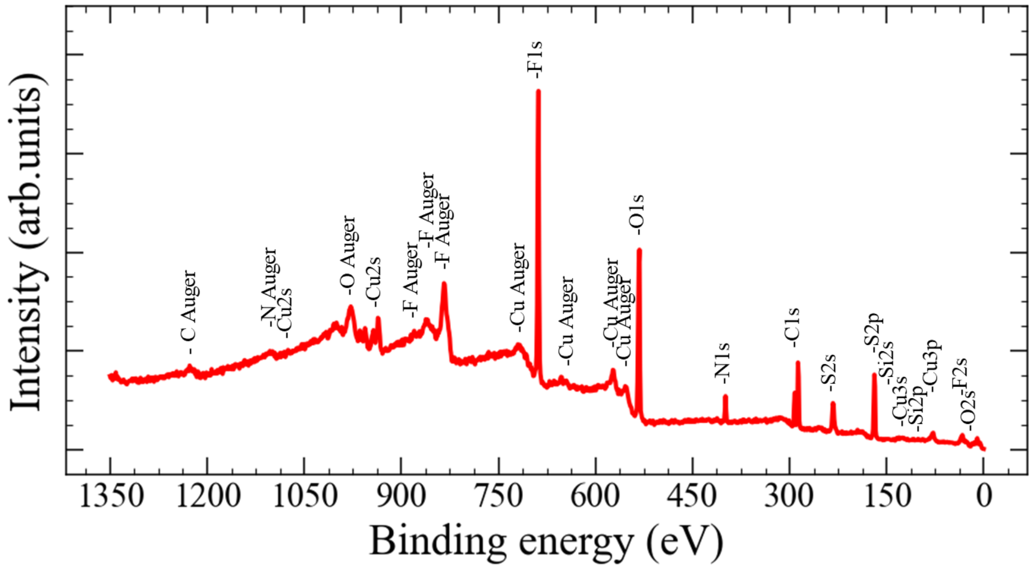

Figure S7 XPS survey spectrum for Cu-G3 and typical peak positions for the detected elements.

To investigate the retention characteristics of the IL-reservoir after the Cu deposits formation on the Pt electrode under different voltage conditions, three samples (S1, S2, and S3) were equivalently prepared on the same substrate. For these devices, after dual voltage sweep with positive bias (0 → +3.0 → 0 V), single voltage sweep with negative bias was performed for each device. The maximum absolute voltage values to which the voltage was swept in the negative voltage sweep process for Devices S1, S2, and S3 were -0.5, -1.5, and -3.0 V, respectively, as shown in Figure S8(a)–(c). Voltage application was stopped immediately after reaching the maximum absolute voltage values, and optical microscopy observations were performed. Figure S9 summarizes the optical microscopy observation results in Devices S1, S2, and S3 immediately after reaching the maximum absolute voltage values (Figure S9(a), (d), and (g), respectively),

after 5 min (Figure S9(b), (e), and (h)), and after 25 min (Figure S9(c), (f), and (i)). The color of the Cu deposit on the left electrode changed from brown with the metallic luster of copper to black over time regardless of the stop voltage. In addition, Cu deposit dissolution was observed, especially on the grounded right Pt electrode. For instance, when Figure S9(g) and (i) for Device S3 are compared, the area of Cu deposit on the grounded electrode (right) clearly decreases. These color changes and deposit dissolution processes can be related with current relaxation processes in the IL-reservoir.

The observed color change might be attributed to the oxidization reaction of the Cu deposit by oxygen and/or H_2O in the air or with those dissolved in Cu-G3, producing CuO, which is well-known for its black color. On the other hand, the dissolution of the Cu deposit can be attributed to the chemical reaction between the Cu deposits and IL or H_2O. Further investigation, such as chemical bonding state evaluation, is necessary for a more precise understanding of the mechanism of the color change and deposit dissolution.

It should be noted that the relaxation time of 5 min is thought to be an overestimated value compared to actual relaxation time because the degree of the color change and the amount of deposit dissolution are required to be large enough to be detected by the optical microscope used in our real-time observation. Actually, electrical measurement results shown in Figure S10 and S11 indicate that the Cu dissolution process starts at much earlier stage than 5 min after the voltage application is stopped. It was revealed that a wait time (T_{wait}) shown in Figure S10, which was introduced between 2nd and 3rd TVP having opposite voltage polarity, strongly influenced the output current for the 3rd TVP. Compared with the case of $T_{wait} = 0$ shown in Figure S11(a), the current peak height for 3rd TVP application already decreased even when $T_{wait} = 10$ s. Generally, the retention characteristics is thought to depend on the voltage condition when the Cu deposit is formed on the Pt electrode. Therefore, by decreasing the voltage application time and/or voltage amplitude for the 1st and 2nd TVP, the retention time shorter than 10 s may be realized. The T_{wait} dependent current signal shown in Figure S11 indicates

that the present IL-reservoir can generate the output signal reflecting the input voltage signal timing, which is a quite advantages property for processing time-series data.

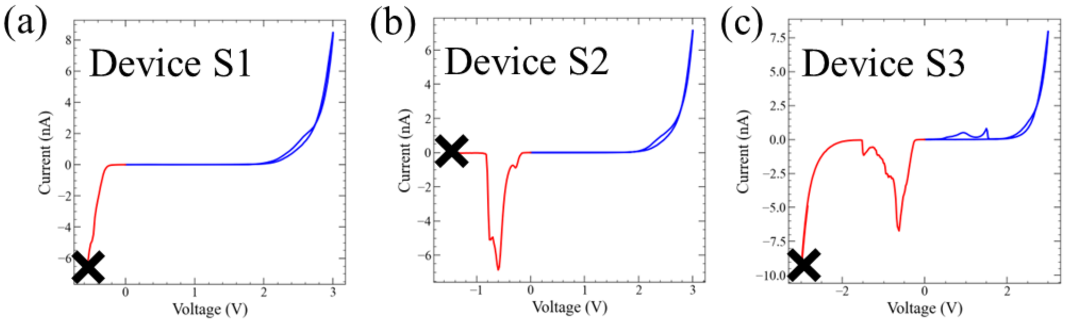

Figure S8 *I-V* profiles of three IL-reservoirs, named (a) S1, (b) S2, and (c) S3, which were equivalently prepared on the same substrate. After dual sweep of voltage with positive bias (0 → +3.0 → 0 V) shown by blue lines, single sweep of voltage with negative bias was performed for each device as shown by red lines. The maximum absolute voltage values to which the voltage was swept in the negative voltage sweep process were -0.5, -1.5, and -3.0 V for Devices S1, S2, and S3, respectively.

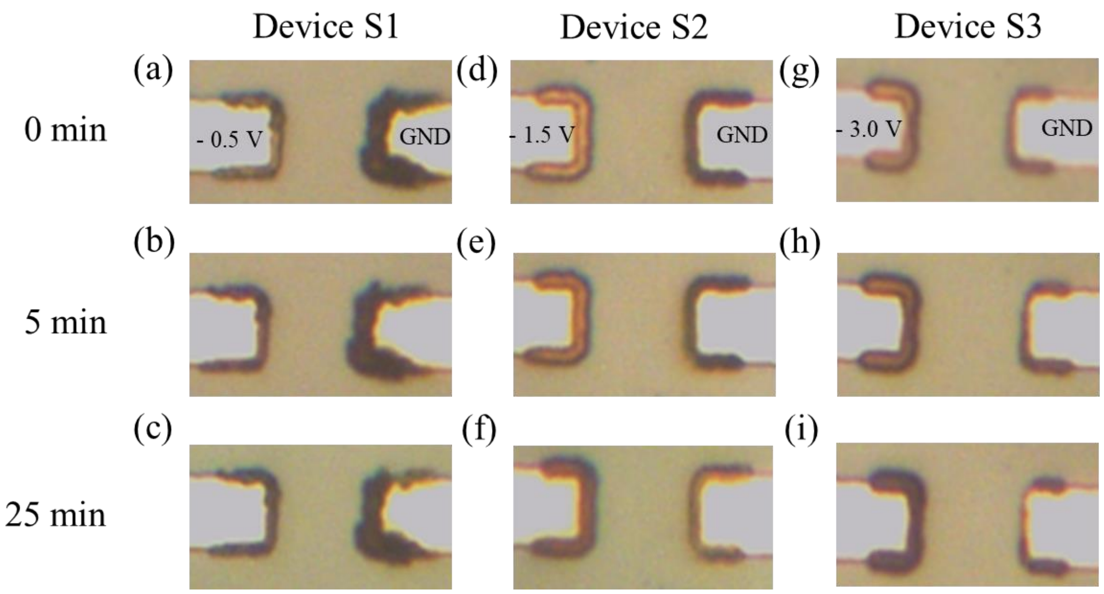

Figure S9 The optical microscopy observation results in Devices S1-3, whose *I-V* profiles until starting the observation are given in Figure S8(a)-(c), immediately after

reaching the maximum absolute voltage values ((a), (d), and (g) respectively for S1, S2, and S3), after 5 min ((b), (e), and (h)), and after 25 min ((c), (f), and (i)).

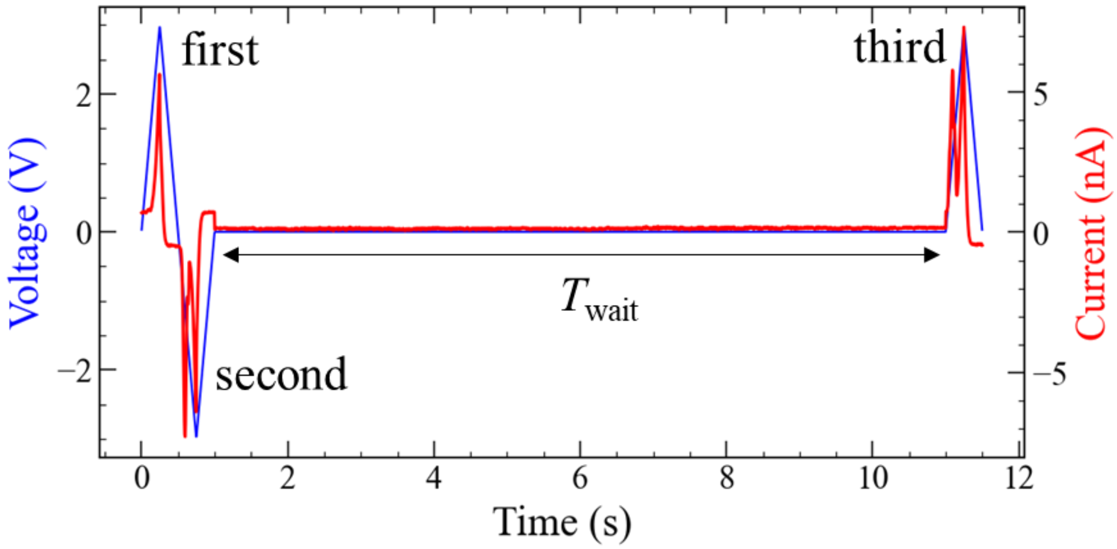

Figure S10 Three input TVPs with the wait time T_{wait} introduced between 2nd and 3rd TVP (left axis) and corresponding output current signal from IL-reservoir (right axis) are shown by blue and red lines, respectively.

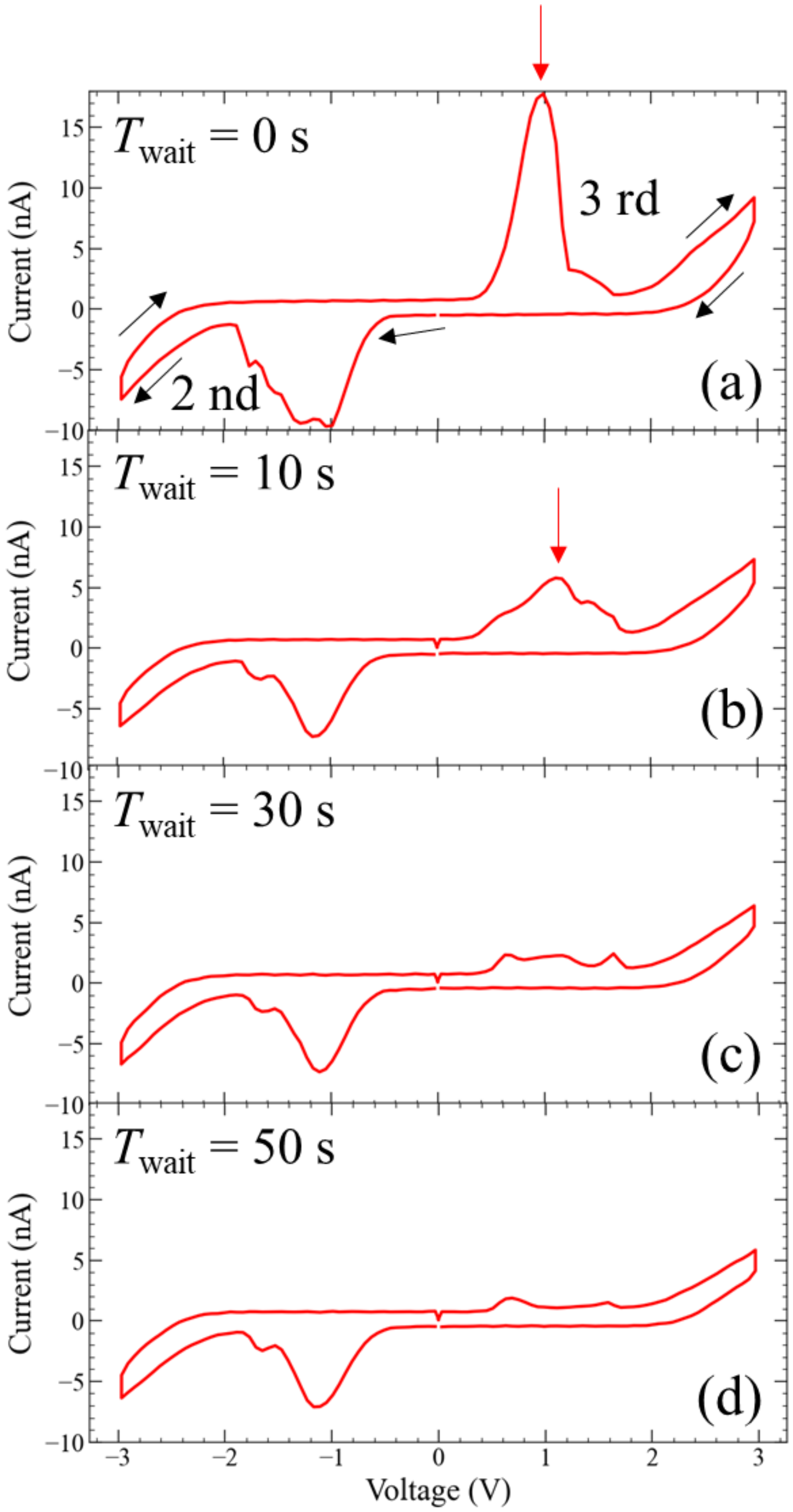

Figure S11 T_{wait} dependence of the *I-V* curve with (a) T_{wait} = 0 s, (b) 10 s, and (c) 30 s, and (d) 50 s. Black arrows indicate voltage sweep direction.

Figure S12 is the time variation in current measured without IL-reservoir. The current level without IL-reservoir, which is regarded as the background current noise level, was approximately 100 pA and sufficiently low to evaluate the electrical property of the IL-reservoir.

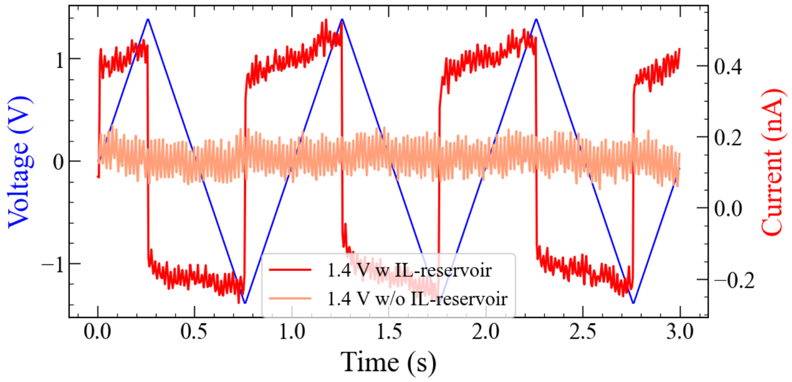

Figure S12 Background current value evaluated using the same measurement condition for the IL-reservoir. The applied triangular voltage pulse (Pulse height: 1.4 V, pulse width: 500 ms) is plotted by the blue line (left axis), while the background current value is plotted by the orange line (right axis). For compassion, the output current value from the IL-reservoir is also plotted by the red line.

We evaluated the cycle dependence of the current response at room temperature (23 °C), which is shown in Figure S13(a). We repeated the *I-V* curve measurement totally 900 times. For the viewability, the *I-V* curves in the 40-50 cycles, 690-700 cycles, and 840-850 cycles are plotted. In this measurement, alternating application of triangular voltage pulses with pulse height of +2.8 V and -2.8 V and pulse width of 500 ms was carried out. Although the current peak intensity decreased with increasing the cycle number (especially after 800 cycles), it still remains in the 900th *I-V* curve. Furthermore, the current values at maximum voltage application (+2.8 V and -2.8 V) also decrease with increasing the cycle number. Those current decrease can be attributed to the Cu ion concentration decrease in the ionic liquid probably due to gradually developing irreversible copper deposition reaction. The cycle stability of our IL-reservoir is affected by some operation conditions such as the operation temperature and pulse width used for the device operation. For instance, as shown in Figure S13(b) the cycle stability at the elevated temperature (at 50 °C) was different from that measured at 23 °C shown in Figure S13(a). Furthermore, as shown in Figure S13(c), more stable cycle endurance characteristics was observed when the pulse width of 5 ms was used instead of 500 ms in Figure S13(a). Although the contribution from the charge/discharge current by the electric double layers (EDLs) to the current value seems to be larger for the pulse width of 5 ms compared to 500 ms, a current peak by the electrochemical reaction is still clearly observed.

In Figure S14(a), we compared the influence of the temperature on the distribution of the current peak values which are extracted from the results in Figure S13. The current dispersion was suppressed by increasing the temperature value. Also, in Figure S14(b), we compared the influence of the voltage pulse width on the distribution of the current peak values. The current dispersion was strongly suppressed by decreasing the voltage pulse width. We consider that the temperature increases and voltage pulse width decrease has the same effect on the amount of the Cu deposit on the Pt electrode. More specifically, the temperature increase promotes the Cu deposit dissolution reaction, while the voltage pulse width decrease suppresses the Cu deposit formation. Namely, those two conditions

are appropriate to initialize the Pt electrode surface state, leading to the quite reproducible electrochemical reaction and consequent highly stable current values. It can be expected that the cycle endurance characteristics are further improved by selecting the optimum voltage sweep speed and temperature conditions.

In the present study, it has been confirmed that the current peak originated short term memory characteristics and nonlinear transformation capability. Therefore, even if the current peak intensity observed at room temperature decreased with increasing the cycle number as shown in Figure S13(a), the machine learning accuracy can be expected to remain high until the current peak completely disappears. The negative impact of the current peak value shift during the physical reservoir calculation on the machine learning accuracy may be reduced by introducing an appropriate countermeasure such as a batch normalization [Tsunegi, S., et al. “Physical reservoir computing based on spin torque oscillator with forced synchronization.” Appl. Phys. Lett. 114, 164101 (2019).].

The number of operation cycle required for the physical reservoir computing may depend on the task to be tested. For example, according to the previous paper [Gartside, Jack C., et al. "Reconfigurable training and reservoir computing in an artificial spin-vortex ice via spin-wave fingerprinting." Nature Nanotechnology (2022): 1-10.], at least 450 training dataset, which corresponds to the 450 cycles of the device operation for our IL-reservoir, is reportedly necessary to obtain sufficient accuracy for the inverse saw wave transformation prediction task.

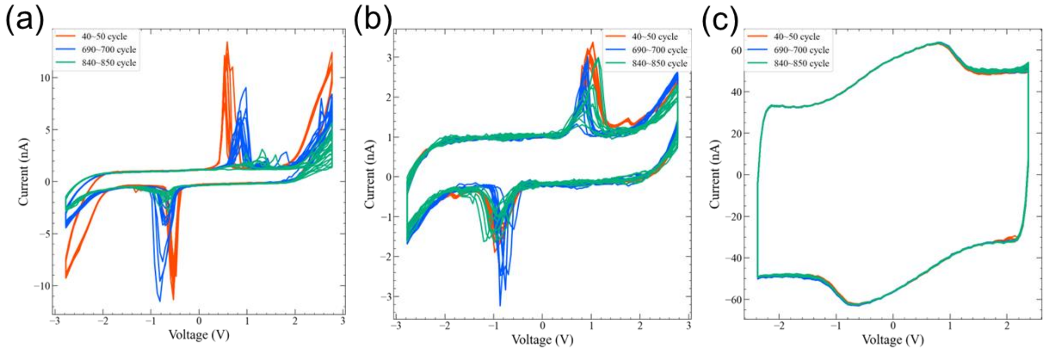

Figure S13 Measurement condition (pulse width P_W and temperature T) dependence of

the *I-V* curve reproducibility for the IL-reservoir evaluated from the 900 times cycle operation results. For viewability, a part of the measured data is used to draw the figure. Measurement condition is (a) P_W = 500 ms, and T = 23 °C, (b) P_W = 500 ms, T = 50 °C, (c) P_W = 5 ms, T = 23 °C.

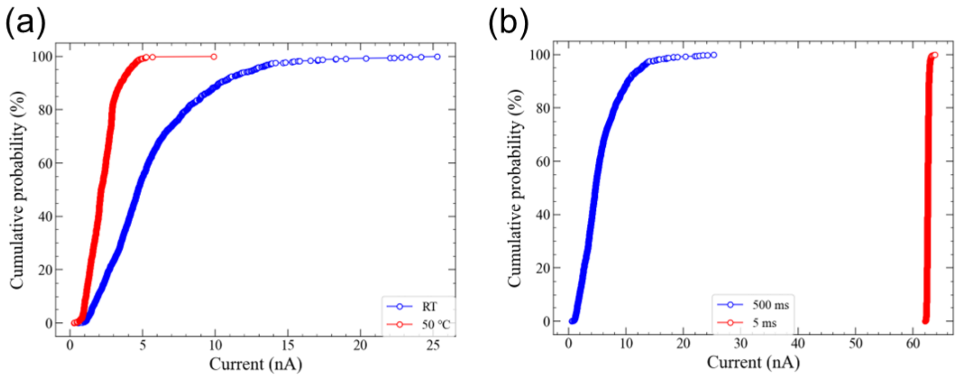

Figure S14 (a) Temperature and (b) pulse width dependence of cumulative probability of the Faradaic current value. Here, the Faradaic current value is determined to be the peak current value around V = 1.0 V in the *I-V* curve in Figure S13 (a) – S13(c).

Figure S15 (a) is the voltage pulse width dependence of the information processing accuracy for the STM tasks. Especially for STM_2, the decrease of the voltage pulse width caused the improvement of the information processing accuracy. As shown in the Figure S13 (c), the decrease of the voltage pulse width suppresses the dispersion of the current value. The current value dispersion often makes the output signal for different input voltage signal more indistinguishable, especially for the large value of T_{delay}. Therefore, by suppressing the current value dispersion, highly reproducible and distinguishable output signal for the identical TVP stream can be obtained, leading to the high accuracy for the STM_2 task.

Figure S15 (b) is the virtual node number N dependence of the information processing accuracy for the STM tasks. When $N = 25$, the virtual nodes were selected every 4th node from 100 nodes in one time step. When $N = 50$, the virtual nodes were selected every 2nd node. For STM_0, there is no influence from the value of N. On the other hands, for STM_1 and STM_2, the decrease of N lowered the information processing ability. The obtained result on the N value dependence indicates that larger number of N is necessary for more complicated information processing task.

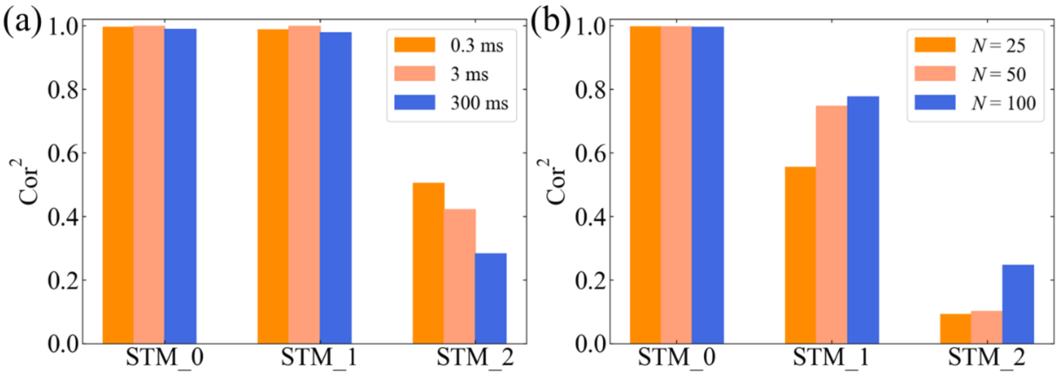

Figure S15 The influence from (a) voltage pulse width of input TVPs and (b) virtual node number (N) on the information processing accuracy for the STM tasks.

As shown in the optical microscope images in Figure S16, we prepared the IL-reservoir devices having different interelectrode distance. The interelectrode distance was varied from 2 μm to 8 μm. The *I-V* characteristics for those devices are shown in Figure S17. There was no clear difference in *I-V* characteristics, which is originated from two factors: electric field concentration on the electric double layer at the IL/electrode interface and the microfabricated device structure in the present IL-reservoir device. Generally, almost all the external electric field concentrates on the IL/electrode interfaces. Besides, since both the electrode area and interelectrode distance in the present IL-reservoir are quite small compared to the electrode geometry in the general electrochemical cell, the voltage drop in the IL region between the electrodes is negligibly small. Therefore, there was no influence of the interelectrode distance on the electrical property of the IL-reservoir. Therefore, we believe that the influence of interelectrode distance on the information-processing performance of reservoir devices is also limited.

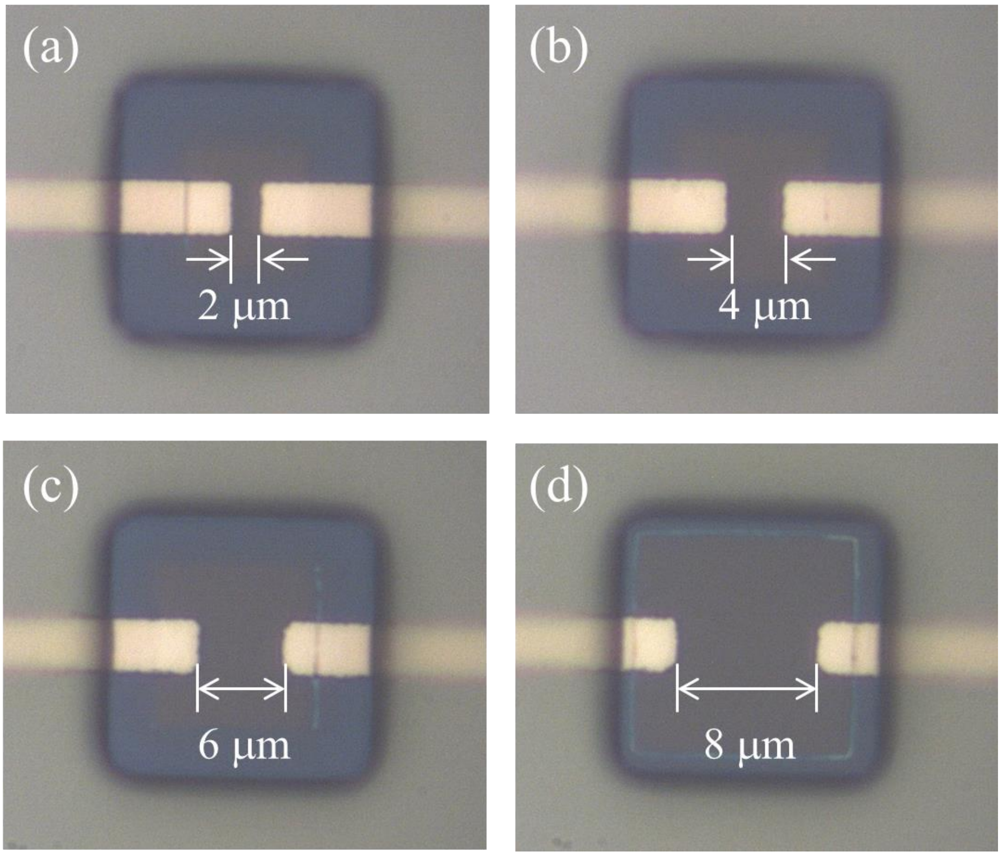

Figure S16 Optical microscope images for the IL-reservoirs to evaluate the influence of the interelectrode distance. The interelectrode distance is (a) 2 μm, (b) 4 μm, (c) 6 μm, and (d) 8 μm.

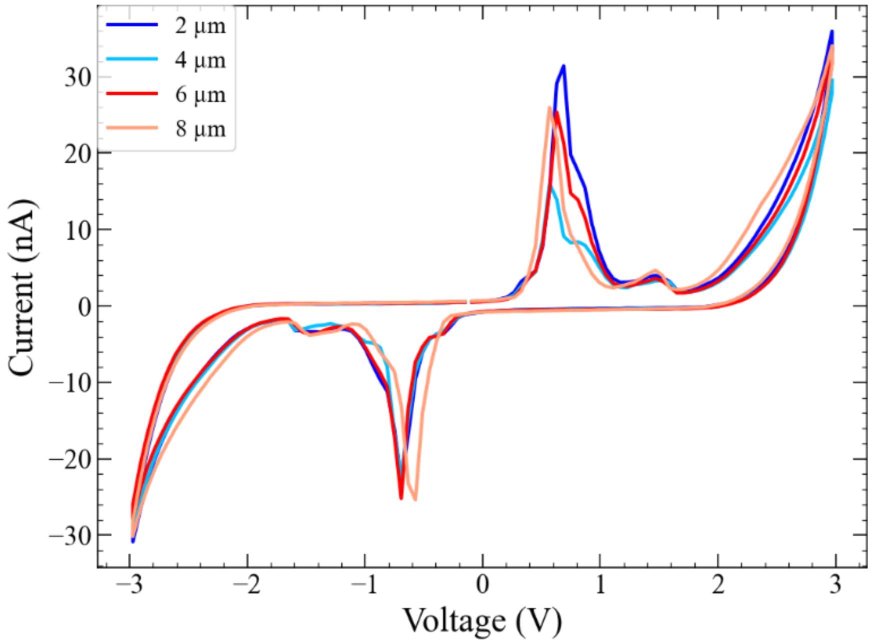

Figure S17 Inter-electrode distance dependence of the *I-V* curves in the present IL-reservoir. The interelectrode distance was varied from 2μm to 8 μm.

We also evaluated the influence of the electrode area. In this case, we used the IL-reservoir devices having a device structure shown in Figure S18, which is suitable for changing dynamically the electrode area of the device. The IL/electrode interfaces are formed in the area surrounded by the red dotted squares, which corresponds to the reaction area in the present IL-reservoir device. In the present study, the reaction area was 10 × 10 μm^2, 100 × 100 μm^2, and 300 × 300 μm^2. The figures S19 (a)-(c) show the current waveforms for the IL-reservoir device having the reaction area of 10 × 10 μm^2, 100 × 100 μm^2, and 300 × 300 μm^2, respectively. At first, in both the voltage sweep directions, the current value around $V = 0$ V increases as the electrode area increases, indicating that the charge/discharge current due to the capacitance component of the electric double layer increases with the electrode area.

In addition, the width of the current peak increases with the electrode area, which indicates that the chemical reaction at the IL/electrode interface proceeds inhomogeneously according to the electrical field distribution in the inside the electrode region. Figures S20 show the electrode area dependence of the STM task accuracy. With

decreasing the electrode area, the STM accuracy decreased, which was probably due to the larger dispersion of current values in the IL-reservoir having smaller electrode area as shown in Figures S19. From the view point of the implementation in the integrated circuits, the establishment of the compatibility between the device size miniaturization and high reliability by the development of IL materials and IL-reservoir device structures is required.

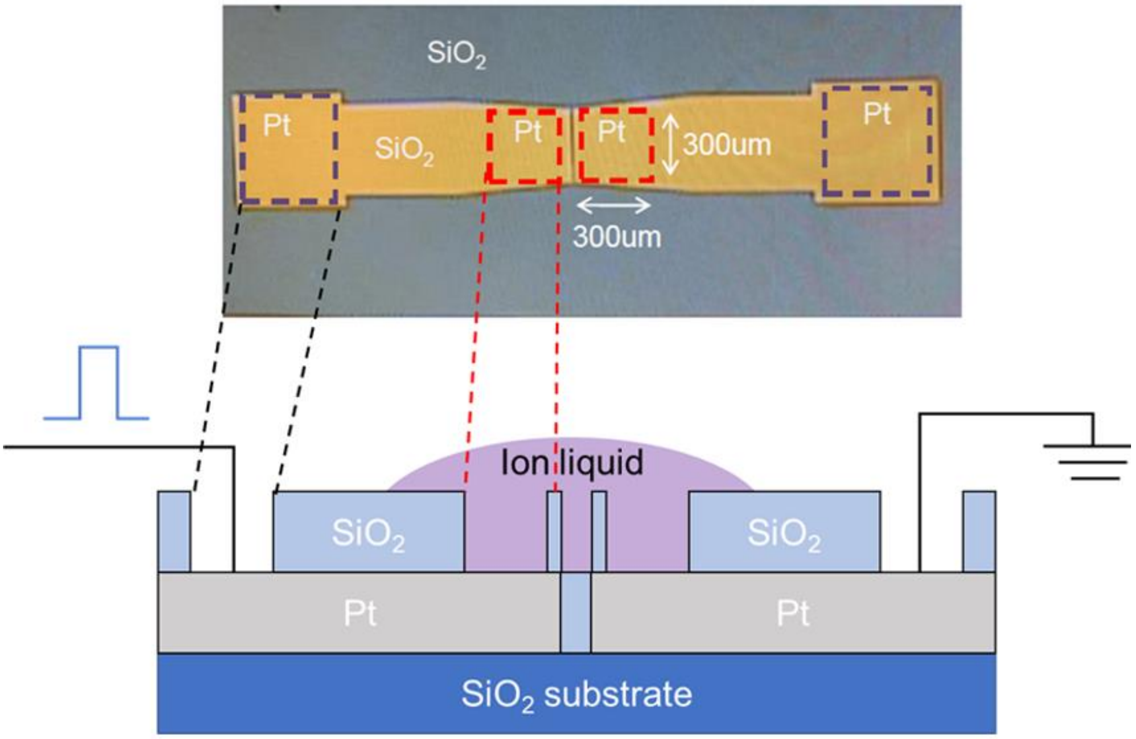

Figure S18 Optical microscope of the device top view and corresponding schematic of the cross-section for the IL-reservoir prepared to evaluate the influence of the electrode area. The electrode areas are 10 × 10 μm^2, 100 × 100 μm^2, and 300 × 300 μm^2. The right electrode is used to apply the voltage, while the left electrode is grounded.

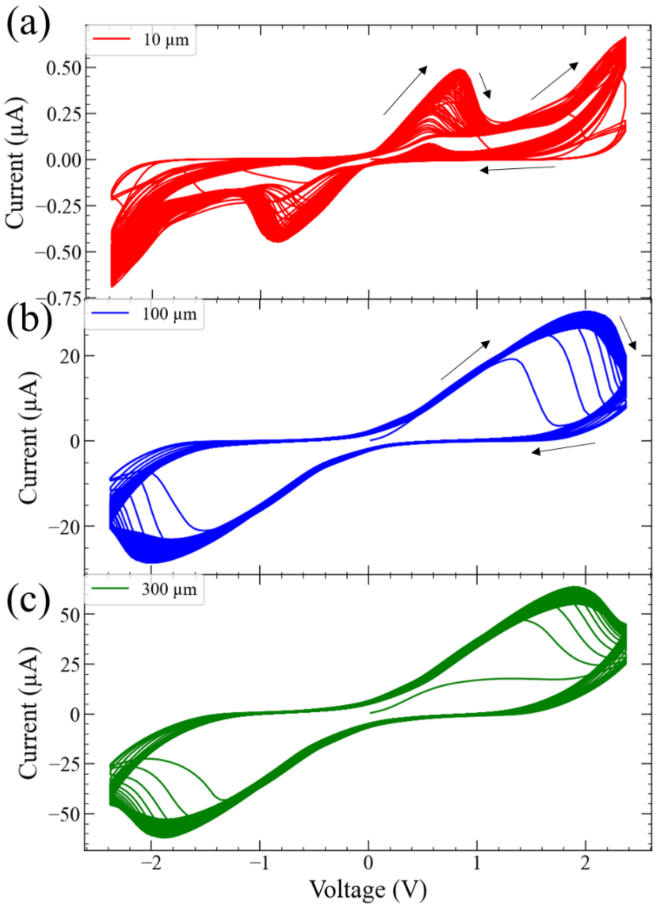

Figure S19 Electrode area dependence of *I*-*V* curve measured using the IL-reservoir shown in Figure S18. Electrode area is (a) 10 × 10 μm^2, (b) 100 × 100 μm^2, and (c) 300 × 300 μm^2.

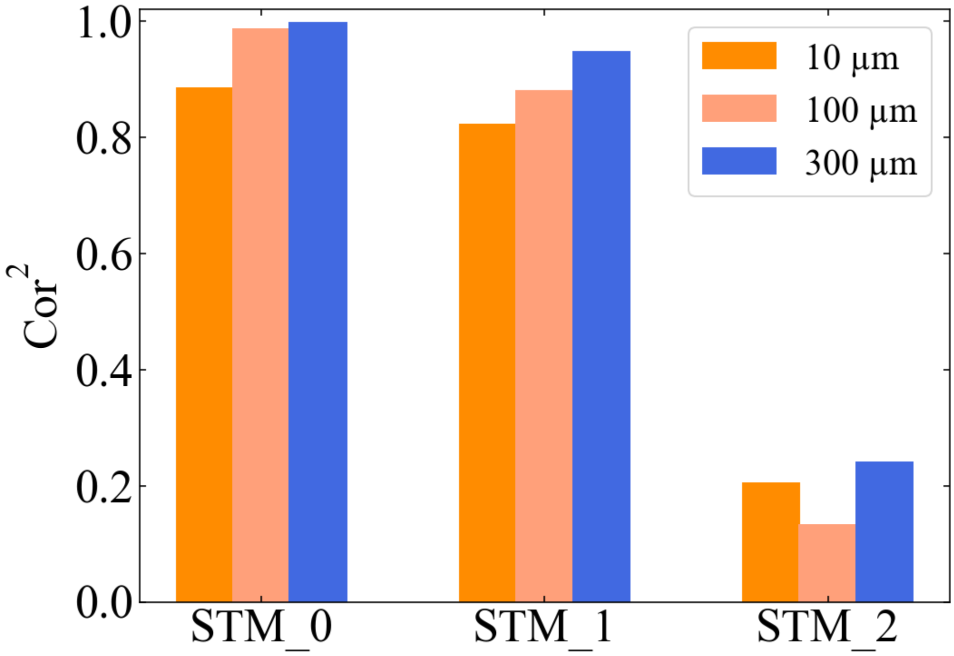

Figure S20 Electrode area dependence of the STM task accuracy. The IL-reservoir device having different electrode area, 10 × 10 μm^2, 100 × 100 μm^2, and 300 × 300 μm^2, were compared.

We compared the machine learning results using SGD shown in Figure 11(c) with that using the other weight update method called Momentum. The equation for Momentum is shown below

$$v = \alpha v - \eta \frac{\partial E}{\partial W} \qquad 1$$

$$W = W + v \qquad 2$$

As shown in Figure S21, almost no influence from the weight update method on the machine learning results was observed.

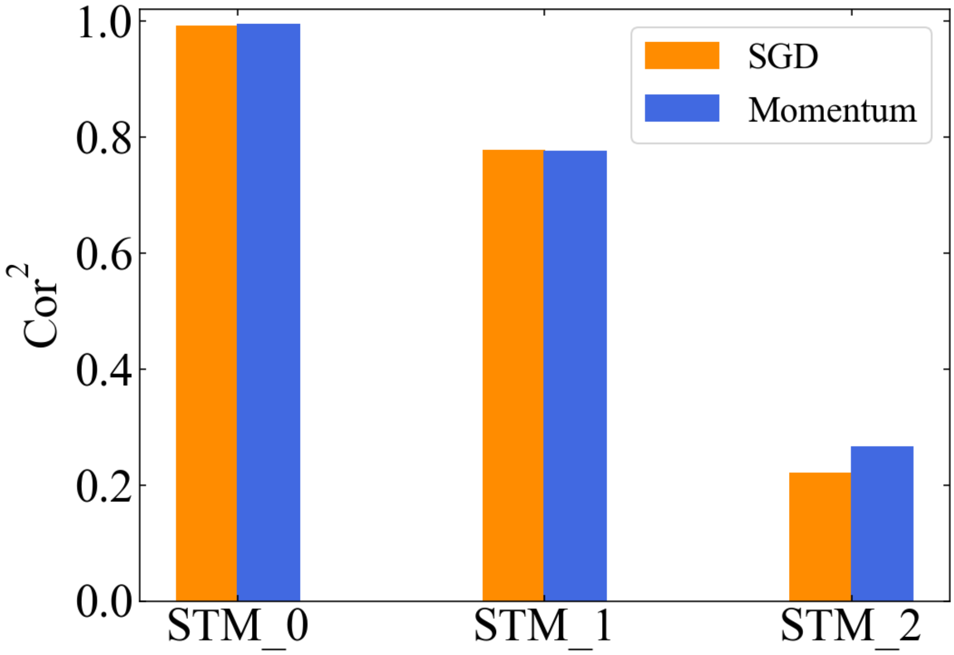

Figure S21 Weight update method dependence of the STM task accuracy (Measurement condition S2). Two weight update method, SGD and Momentum, were compared.

Table S2 The detail on the evaluated correlation coefficient values (Cor) for the STM tasks shown Figure 11a-c. Each calculation was conducted 3 times and averaged values (highlighted in red) were used to for the bar chart in Figure 11a-c.

Cor			STM_0				STM_1				STM_2			
			1st run	2nd run	3rd run	Average	1st run	2nd run	3rd run	Average	1st run	2nd run	3rd run	Average
Figrue 11a	dataset-F	S1	0.996	0.991	0.996	0.994	0.866	0.868	0.867	0.867	0.226	0.237	0.252	0.239
		S2	0.998	0.995	0.997	0.997	0.854	0.844	0.859	0.852	0.455	0.501	0.460	0.472
		A1	0.997	0.995	0.995	0.996	0.877	0.883	0.895	0.885	0.535	0.545	0.526	0.535
		A2	0.993	0.988	0.990	0.990	0.788	0.805	0.808	0.800	0.583	0.568	0.593	0.582
Figrue 11b	dataset-L	S1	0.996	0.996	0.997	0.997	0.724	0.680	0.708	0.704	0.255	0.297	0.283	0.278
		S2	0.999	0.999	0.999	0.999	0.668	0.661	0.652	0.660	0.110	0.074	0.103	0.096
		A1	0.999	0.999	1.000	0.999	0.358	0.361	0.358	0.359	0.247	0.242	0.250	0.247
		A2	1.000	1.000	0.999	1.000	0.231	0.257	0.249	0.246	0.013	0.017	0.020	0.017
Figrue 11c	dataset-A	S1	0.988	0.991	0.988	0.989	0.840	0.880	0.808	0.842	0.350	0.464	0.520	0.444
		S2	0.997	0.997	0.997	0.997	0.876	0.886	0.882	0.881	0.498	0.493	0.468	0.486
		A1	0.998	0.995	0.994	0.996	0.889	0.888	0.902	0.893	0.528	0.539	0.533	0.533
		A2	0.999	0.998	0.997	0.998	0.908	0.922	0.910	0.913	0.597	0.586	0.562	0.582

Table S3 The detail on the evaluated correlation coefficient values (Cor) for the PC tasks shown Figure 11d-f. Each calculation was conducted 3 times and averaged values (highlighted in red) were used to for the bar chart in Figure 11d-f.

Cor			PC_1				PC_2			
			1st run	2nd run	3rd run	Average	1st run	2nd run	3rd run	Average
Figrue 11d	dataset-F	S1	0.212	0.230	0.230	0.224	0.633	0.632	0.647	0.637
		S2	0.179	0.182	0.177	0.179	0.540	0.565	0.557	0.554
		A1	0.884	0.875	0.889	0.883	0.533	0.526	0.537	0.532
		A2	0.919	0.916	0.911	0.916	0.518	0.489	0.523	0.510
Figrue 11e	dataset-L	S1	0.166	0.103	0.187	0.152	0.643	0.672	0.614	0.643
		S2	0.186	0.182	0.186	0.185	0.523	0.543	0.532	0.533
		A1	0.879	0.880	0.875	0.878	0.555	0.540	0.548	0.548
		A2	0.913	0.935	0.907	0.918	0.507	0.514	0.504	0.508
Figrue 11f	dataset-A	S1	0.144	0.215	-0.045	0.105	0.434	0.577	0.599	0.537
		S2	0.183	0.173	0.182	0.179	0.554	0.556	0.540	0.550
		A1	0.894	0.897	0.901	0.898	0.543	0.538	0.546	0.543
		A2	0.922	0.915	0.918	0.918	0.532	0.547	0.531	0.537
